# Association of Dance-Based Mind-Motor Activities With Falls and Physical Function Among Healthy Older Adults: A Systematic Review and Meta-analysis

**DOI:** 10.1001/jamanetworkopen.2020.17688

**Published:** 2020-09-25

**Authors:** Michèle Mattle, Patricia O. Chocano-Bedoya, Melanie Fischbacher, Ursina Meyer, Lauren A. Abderhalden, Wei Lang, Richard Mansky, Reto W. Kressig, Johann Steurer, E. John Orav, Heike A. Bischoff-Ferrari

**Affiliations:** 1 Department of Geriatrics and Aging Research, University Hospital Zurich and University of Zurich, Zurich, Switzerland; 2 Center on Aging and Mobility, University of Zurich, Zurich, Switzerland; 3 University Department of Geriatric Medicine Felix Platter, Basel, Switzerland; 4 Horten Centre, University of Zurich, Zurich, Switzerland; 5 Department of Biostatistics, Harvard T.H. Chan School of Public Health, Boston, Massachusetts

## Abstract

**Importance:**

Falls increase morbidity and mortality in adults 65 years and older. The role of dance-based mind-motor activities in preventing falls among healthy older adults is not well established.

**Objective:**

To assess the effectiveness of dance-based mind-motor activities in preventing falls.

**Data Sources:**

Systematic search included the PubMed, Embase, Cochrane Library, Web of Science, CINAHL, PsychINFO, Abstracts in Social Gerontology, AgeLine, AMED, and Scopus databases from database inception to February 18, 2018, using the Medical Subject Headings *aged 65 and older*, *accidental falls*, and *dancing*.

**Study Selection:**

This systematic review and meta-analysis included 29 randomized clinical trials that evaluated a dance-based mind-motor activity in healthy older adults with regard to fall risk, fall rate, or well-established measures of physical function in the domains of balance, mobility, and strength. The included studies targeted participants without comorbidities associated with higher fall risk. Dance-based mind-motor activities were defined as coordinated upright mind-motor movements that emphasize dynamic balance, structured through music or an inner rhythm (eg, breathing) and distinctive instructions or choreography, and that involve social interaction.

**Data Extraction and Synthesis:**

Standardized independent screening, data extraction, and bias assessment were performed. Data were pooled using random-effects models. The study followed the Preferred Reporting Items for Systematic Reviews and Meta-analyses (PRISMA) guideline.

**Main Outcomes and Measures:**

Primary outcomes were risk of falling and rate of falls. For the secondary end points of physical function (balance, mobility, and strength), standardized mean differences (SMDs) were estimated and pooled (Hedges *g*).

**Results:**

In this systematic review and meta-analysis of 29 randomized clinical trials, dance-based mind-motor activities were significantly associated with reduced (37%) risk of falling (risk ratio, 0.63; 95% CI, 0.49-0.80; 8 trials, 1579 participants) and a significantly reduced (31%) rate of falls (incidence rate ratio, 0.69; 95% CI, 0.53-0.89; 7 trials, 2012 participants). In addition, dance-based mind-motor activities were significantly associated with improved physical function in the domains of balance (standardized mean difference [SMD], 0.62; 95% CI, 0.33-0.90; 15 trials, 1476 participants), mobility (SMD, −0.56; 95% CI, −0.81 to −0.31; 13 trials, 1379 participants), and lower body strength (SMD, 0.57; 95% CI, 0.23-0.91; 13 trials, 1613 participants) but not upper body strength (SMD, 0.18; 95% CI, −0.03 to 0.38; 4 trials, 414 participants).

**Conclusion and Relevance:**

Among healthy older adults, dance-based mind-motor activities were associated with decreased risk of falling and rate of falls and improved balance, mobility, and lower body strength. This type of activity may be useful in preventing falls in this population.

## Key Points

**Question:**

Are dance-based mind-motor activities associated with benefits for fall prevention and better physical functions, such as balance, mobility, and strength, in healthy adults 65 years and older?

**Findings:**

This systematic review and meta-analysis of 29 randomized clinical trials found that dance-based mind-motor interventions were associated with a statistically significant reduction (37%) in fall risk and a statistically significant reduced rate (31%) of falls. There was a statistically significant association between favorable physical function outcomes and dance-based mind-motor activities for participants in the dance-based mind-motor intervention groups compared with those in the control groups.

**Meaning:**

Dance-based mind-motor activities may help fall prevention efforts in healthy older adults.

## Teaser

This systematic review and meta-analysis assesses the association of dance-based mind-motor activities with falls and physical function among healthy older adults.

## Introduction

In industrialized countries, life expectancy and the number of age-related chronic diseases are increasing.^1^ Currently, approximately 30% of community-dwelling adults 65 years and older experience a fall per year, increasing up to approximately 50% of adults 80 years and older.^2,3,4^ Notably, more than 30% of falls among older adults need medical attention, and approximately 5% to 7% of falls result in a fracture.^4,5^ Consequently, falls are costly^6^ and carry a high risk of functional decline and loss of autonomy for the individual.^4^

On the other hand, exercise interventions are effective in improving strength, balance, and gait and reducing falls and related injuries among older adults.^7,8^ Dance-based mind-motor activities have been suggested as physical exercise^9,10,11^ with extended benefits beyond the physical on cognition,^12,13^ social interaction,^14,15^ quality of life,^16^ and motivation to be physically active.^17,18^ Mechanistically, these benefits may be explained by the multidimensional nature of these activities, which combine sensorimotor and cognitive engagement,^19,20^ relevant to fall prevention.

To our knowledge, no prior meta-analysis of randomized clinical trials (RCTs) has reported on associations between dance-based mind-motor activities and the risk of falling, the rate of falls, and physical function outcomes (balance, mobility, and strength). Therefore, we conducted a systematic review and meta-analysis to summarize the current evidence from RCTs among healthy adults 65 years and older.

## Methods

### Data Sources and Searches

For this systematic review and meta-analysis, we conducted a systematic search in PubMed, Embase, the Cochrane Library, Web of Science, CINAHL, PsychINFO, Abstracts in Social Gerontology, AgeLine, AMED, and Scopus databases from database inception to February 18, 2018, using thesaurus terms and key words (eAppendix in the Supplement for search terms used in PubMed). In addition, we performed lateral screening of the bibliographies of reviewed publications. We contacted authors of articles without sufficient information for complete data.

### Study Selection

Screening and data extraction were predefined and standardized and followed the Preferred Reporting Items for Systematic Reviews and Meta-analyses (PRISMA) reporting guideline.^21^ At every stage, 2 independent researchers (from among M.M., M.F., U.M., and R.M.) screened each article. Disagreements were solved by consensus and, if necessary, by the consultation of a third independent reviewer (P.O.B.-C.). We included only RCTs that analyzed the effect of dance-based mind-motor activities on the number of persons who fell (risk of falling) or on the number of falls (rate of falls) as primary outcomes or at least 1 of the selected secondary outcomes related to physical function (balance, mobility, or strength) compared with an active (exercise not related to dance-based mind-motor activities) or a passive control group.

We defined dance-based mind-motor activities as coordinated upright mind-motor movements that emphasize dynamic balance, structured through music or an inner rhythm (eg, breathing) and distinctive instructions or choreography, and involve social interaction. Mind-motor activities are activities that combine cognitive and physical tasks (dual tasking or multitasking activities) that involve working memory and deliberate motor control. Dance-based mind-motor activities can be performed solo, in a pair, or in group formations. In addition to several styles of well-known dance-based mind-motor activities, such as folk or ballroom dancing, tai chi fulfills the above definition. Contrarily, most yoga styles focus on static body postures rather than dynamic movements and therefore were excluded. Furthermore, we excluded stepping exercises on so-called dance platforms because of the lack of social interaction.

We only considered RCTs that included healthy and ambulatory older adults (mean age, ≥65 years) living in the community or in independent living facilities. We excluded studies involving participants with comorbidities that directly affect the risk of falling (eg, Parkinson disease, type 2 diabetes, and cognitive decline). Only articles in the English language fulfilled the eligibility criteria. Two reviewers (from among M.M., M.F., U.M., and P.O.B.-C.) independently assessed potential sources of bias using the Cochrane Collaboration’s tool for assessing risk of bias.^22^ All included studies had been approved by an ethics committee and informed consent of participants had been obtained.

### Statistical Analysis

To determine the strength of associations between a dance-based mind-motor activity and risk of falling and rate of falls, we pooled risk ratios (RRs) for risk of falling and Poisson-derived incidence rate ratios (IRRs) for the rate of falls using random-effects models based on intention-to-treat analysis. We estimated the IRRs based on reported incident falls and person-years in trials that did not directly report IRRs.

For the secondary outcomes, only trials that reported standardized, validated, and clinically used tests to measure balance, mobility, or strength were included: the Berg Balance Scale,^23,24^ the 1-leg test,^25,26^ and the functional reach test^27^ for balance; Timed Up and Go test^28^ for mobility; and sit to stand tests^29,30^ for lower body strength and the handgrip strength test^31^ for upper body strength. To determine the strength of associations between a dance-based mind-motor activity and physical function measures, we calculated a pooled Hedges *g* standardized mean difference (SMD) using a random-effects model.^32^ If a study reported outcomes stratified or by 2 tests that assessed the same outcome, we pooled the reported effect sizes before entering them into the meta-analysis. We weighted stratified outcomes by the inverse of the variance, assumed dependency between the assessments, and used a conservative correlation coefficient of 0.8.^32^ To interpret the effect of the intervention, we followed the guideline suggested by Cohen: equivalent effect size (SMD of 0), small effect size (SMD of 0.2), medium effect size (SMD of 0.5), and large effect size (SMD of 0.8).^33^

Heterogeneity was quantified by the *I*^2^ statistic as suggested by Borenstein et al.^32^ We performed prespecified subgroup analyses by type of dance-based mind-motor intervention (non–tai chi vs tai chi), frequency (<3 vs ≥3 times per week), duration (<12 vs ≥12 weeks), type of dwelling (community dwelling vs independent living facilities), and type of randomization (participants randomized vs cluster randomization). In addition, we performed a random-effects meta-regression by intervention frequency and intervention duration if more than 10 RCTs were included.

Small-study effects were assessed for each outcome if more than 10 RCTs were available. We conducted the Harbord modified test for the primary outcome of RR of falls^34^ and the Egger test for balance, mobility, and lower body strength.^32^ We used the Egger publication bias plot and funnel plots to visually assess indication of publication bias.^35^ We used Stata software, version 15 (StataCorp LLC) for data analysis.^36^ Statistical assessment was 2 sided and considered statistically significant at *P* < .05.

## Results

### Study Characteristics

From 4627 screened publications, 29 RCTs that fulfilled the inclusion criteria were identified (eAppendix in the Supplement). Eight trials^37,38,39,40,41,42,43,44^ reported on the risk of falling, 7 trials^40,41,42,43,45,46,47^ reported on the rate of falls, and 4 trials reported on both. A total of 15 trials^37,38,39,40,41,44,48,49,50,51,52,53,54,55,56^ reported on balance, 13 trials^39,40,41,45,48,50,54,57,58,59,60,61,62^ reported on mobility, 13 trials^38,45,46,50,55,56,58,59,60,61,63,64,65^ reported on lower body strength, and 4 trials^44,47,55,59^ reported on upper body strength. Table 1 gives an overview of all included trials. Table 2 lists the main descriptive statistics by outcome.

**Table 1.** Characteristics of Included Trials^a^

Source	Population^b^	Activity (No. of participants)			Female, %/age, mean (SD), y		Intervention group			Allocation/blinding of assessors	End point assessment
		Intervention group	Control group	Total	Intervention group	Control group	Frequency, times per wk/class time, min	Duration of intervention	Adherence, mean, %		
Alves et al,^48^ 2013	Community dwelling; members of a dance project sponsored by the government; no further statement about socioeconomic status or race/ethnicity	Ballroom dance (25)	Control (25)	50	84/69 (7)	96/68 (8)	2/120	16 wk	90	Participants randomized/NR or unclear	Balance: BBS; mobility: TUG test
Bennett et al,^49^ 2018	Community dwelling; recruited from the northwest Florida community via local senior centers; 91% White, 8.7% Black; most participants with grades 11-12 education	Line dancing (12)	Usual care/no exercise (11)	23	83/73 (8) (total, both groups combined)	91	2/60	8 wk	80	Participants randomized/no blinding of assessors	Balance: BBS
Cepeda et al,^57^ 2015	Community dwelling; no further statement about socioeconomic status or race/ethnicity	Ballroom dance (19)	Control (15)	24	100/69 (7)	100/72 (7)	3/60	8 wk	91	Participants randomized/NR or unclear	Mobility: TUG test
Choi et al,^37^ 2004	Institutionalized; no further statement about socioeconomic status or race/ethnicity	Tai chi with music (29)	Control (30)	59	79/77 (8)	70/79 (7)	3/35	12 wk	70	Facilities randomized (cluster randomization)/no blinding of assessors	Falls: risk of falling (RR); weekly monitoring of fall episodes during 12 wk of intervention; balance: OLS eyes open/eyes closed combined
Chyu et al,^45^ 2010	Community dwelling; postmenopausal women; no further statement about socioeconomic status or race/ethnicity	Tai chi (26)	Control (28)	54	100/72 (6)	100/71 (6)	3/60	24 wk	94	Participants randomized/assessors blinded	Falls: rate of falls (IRR); self-reported at baseline, 12 and 24 wk; mobility: TUG test; lower body strength: 5 times STS test
Cruz-Ferreira et al,^58^ 2015	Community dwelling; women recruited from a local health center; no further statement about socioeconomic status or race/ethnicity	Creative dance (32)	Control (25)	57	100/71 (4)	100/73 (5)	3/50	24 wk	85	Participants randomized/assessors blinded	Mobility: TUG test; lower body strength: 30-s STS test
Eyigor et al,^38^ 2009	Community dwelling; recruited in outpatient clinics; in the intervention group 64.7% had primary school education, in the control group 38.5%; most participants were housewives	Turkish folk dance (19)	Control (18)	37	100/74 (8)	100/71 (6)	3/60	8 wk	NR	Participants randomized/assessors blinded	Falls: risk of falling (RR); unpublished data, reported by author on request; balance: BBS; lower body strength: 5 times STS test
Frye et al,^59^ 2007	Community dwelling; 94.4% White/non-Hispanic, 2.8% Black/non-Hispanic, 2.8% Asian/Pacific Islander; well-educated sample: 18.1% with postcollege degree, 20.8% with 4-y college degree, 26.4% with some college degree, 18.1% with high school diploma, 2.8% some high school	Tai chi (23)	Control (21)	44	64/69 (9) (total, both groups combined)		3/60	12 wk	91.4% Of participants attended at least 80% of classes	Participants randomized/PI, project coordinator, and instructors not blinded, unclear if assessors were blinded	Mobility: TUG test; lower body strength: 30-s STS test; upper body strength: HGS
Hopkins et al,^53^ 1990	Community dwelling; no further statement about socioeconomic status or race/ethnicity	Low-impact aerobic dance (30)	Control (23)	53	100/65 (4)	100/66 (4)	3/50	12 wk	NR	Participants randomized/NR or unclear	Balance: OLS; mobility: TUG test; lower body strength: 30-s STS test
Huang et al,^39^ 2010	Community dwelling; 35.5% in tai chi group and 68.1% in the control group had ≤6 y of education	Tai chi (31)	Control (47)	78	29/71 (0)	40/72 (1)	3/40	5 mo (21 wk)	NR	Villages randomized (cluster randomization)/NR or unclear	Falls: risk of falling (RR); assessed during follow-up, means of assessment NR; balance: FR; mobility: TUG test
Hui et al,^60^ 2009	Community dwelling; no further statement about socioeconomic status or race/ethnicity	Low-impact aerobic dance (52)	Control (45)	97	96/68 (5)	98/69 (4)	Approximately 2 (total 23 sessions)/50-60	12 wk	91.3	Social centers randomized (cluster randomization)/assessors blinded	Mobility: TUG test; lower body strength: 10-s STS test (treated same way in meta-analysis as trials reporting 30-s STS test)
Janyacharoen et al,^63^ 2013	Community dwelling; no further statement about socioeconomic status or race/ethnicity	Traditional Thai dance (20)	General aerobic exercises in daily life (18)	38	100/67 (6)	100/65 (4)	3/40	6 wk	NR	Participants randomized/assessors blinded	Lower body strength: 5 times STS test
Li et al,^40^ 2005	Community dwelling; recruited from the pool of patients enrolled in the Legacy Health System in Portland, Oregon; 90% in tai chi group and 91% in control group were White; 94% in tai chi group and 90% in control group had high school degree or higher; annual household income was <$35 000 for 64% in the tai chi group and for 70% in the control group	Tai chi (125)	Seated stretching exercises (131)	256	70/77 (5)	70/78 (5)	3/60	26 wk	Median adherence rate for both groups: 61 of approximately 72 sessions scheduled	Participants randomized/assessors blinded	Falls: risk of falling (RR), fall rate (IRR); means of falls assessment NR; balance: BBS, OLS, FR, combined; mobility: TUG test
Li et al,^51^ 2008	Community dwelling; participating in social and recreational activities outside their home on a minimum of 2 occasions per week; no further statement about socioeconomic status or race/ethnicity	Tai chi (22)	Discussion meetings (18)	40	50/65 (3)	50/66 (4)	4/60	16 wk	92	Participants randomized/NR or unclear	Balance: OLS
Logghe et al,^52^ 2009	Community dwelling; identified through the patient registration files of participating GPs; GPs invited participants to participate; 71% in tai chi group and 65.5% in control group had high school education or more; 96.2% in the tai chi group and 91.3% in the control group were born in the Netherlands	Tai chi (138)	Control (131)	269	70/78 (5)	73/77 (5)	2/60	13 wk	47% Of participants attended at least 80% of classes	Participants randomized/assessors blinded	Balance: BBS
McKinley et al,^64^ 2008	Community dwelling; no further statement about socioeconomic status or race/ethnicity	Tango dance program (14)	Walking (11)	25	78/78 (8)	72/75 (8)	2/120	10 wk	>90 (Both groups)	Participants randomized/assessors blinded	Lower body strength: 5 times STS test
Merom et al,^46^ 2016	Residents of self-care retirement villages; 17% non-English speaking, 73% born in Australia; 36% with ≤10 y of education	Folk or ballroom dance (275)	Delayed intervention (247)	522	83/43% >80 y; all participants at least 60 y	86/35% >80 y; all participants at least 60 y	2/60	12 mo (52 wk/total of 80 lessons)	51	Retirement villages randomized (cluster randomization)/assessors were not blinded at follow-up assessments	Falls: rate of falls (IRR); daily self-report in diaries that were sent to study center on monthly basis; if a fall happened or the diary was not sent in, participants were called to assess details; strength: 5 times STS test
Merom et al,^65^ 2016	Community dwelling; 45.0% in the dance group and 52.7% in the control group had primary/high school as highest education level, 21.7% in the dance group and 20.0% in the control group had TAFE apprenticeship and 33.3% in the dance group, and 27.3% in the control group had university degrees	Ballroom dance (40)	Walking program (39)	79	85/60-69 y: 50%; 70-74 y: 25%; ≥75 y: 25%	85/60-69 y: 62%; 70-74 y: 18%; ≥75 y: 21%	2/60	8 mo (34 wk; approximately 69 sessions)	66	Participants randomized/assessors only at baseline blinded, but not on follow-up assessments	Lower body strength: 5 times STS test
Noradechanunt et al,^61^ 2017	Community dwelling; 76.9% in tai chi group and 92.3% in control group had high school or higher education	Tai chi (9)	Telephone counseling (10)	19	69/67 (8)	77/65 (7)	2/90	12 wk	85	Participants randomized/assessors blinded	Mobility: TUG test; lower body strength: 30-s STS test
Pereira et al,^53^ 2008	Community dwelling; women registered in the university’s physical activity program for elderly people; no further statement about socioeconomic status or race/ethnicity	Tai chi (38)	Control (39)	77	100/68 (5)	100/69 (7)	3/50	12 wk	NR	Participants randomized/NR or unclear	Balance: OLS
Serano-Guzmann et al,^54^ 2016	Community dwelling; White postmenopausal women attending a primary care clinic and referred to the clinical laboratory of the physiotherapy department at the University of Granada, Spain	Flamenco and sevillanas (27)	Self-care treatment advice (25)	52	100/69 (4)	100/69 (3)	3/50	8 wk	100	Participant randomized/assessors blinded	Balance: OLS; mobility: TUG test
Sun et al,^55^ 2015	Community dwelling; recruited via public announcement, 55.5% in the tai chi group and 72.8% in the control group had ≥12 y of education	Tai chi (72)	Social activities (66)	138	81/68 (6)	70/70 (6)	2/60	6 mo (26 wk)	NR	Participant randomized/NR or unclear	Balance: OLS; upper body strength: HGS (results were combined for right and left hand before entering the meta-analysis)
Taylor et al,^62^ 2012	Community dwelling; recruited in Auckland, Dunedin, and Christchurch, New Zealand; 12 participants identified as Māori or Pacific Islander	Group 1: Tai chi (233); group 2: tai chi (220)	Group 1: low-level exercise (231); group 2: low-level exercise (231)	Group 1: 264; group 2: 251	Group 1: 69/75 (7); group 2: 76/74 (6)	Group 1: 76; 74 (6); group 2: 76/74 (6)	Group 1: 1/60; group 2: 2/60	Group 1: 20 wk; group 2: 20 wk	Group 1: 79; group 2: 72	Participants randomized/assessors blinded	Mobility: TUG test (results were combined for groups 1 and 2 before entering the meta-analysis); lower body strength: 30-s STS test (results were combined for groups 1 and 2 before entering the meta-analysis)
Taylor-Piliae et al,^56^ 2010	Community dwelling; recruited in Santa Clara County or San Mateo County in California; college educated, 85% White	Tai chi (37)	Attendance control/no exercise (56)	93	65/71 (6)	73/68 (6)	2/45	6 mo (26 wk)	77	Participants randomized/assessors blinded	Balance: OLS, FR; lower body strength: 30-s STS test
Trombetti et al,^41^ 2011	Community dwelling; 11% in early intervention and 19% in delayed intervention had primary school education, 66% in early intervention and 68% in delayed intervention had middle school education, and 21% in early intervention and 15% in delayed intervention had high school education	Dalcroze eurhythmics (66)	Delayed intervention (68)	134	97/75 (8)	96/76 (6)	1/60	25 wk	83	Participants randomized/assessors blinded	Falls: risk of falling (RR), rate of falls (IRR); prospectively monitored daily with falls diaries that were mailed monthly to study coordinator; balance: OLS; mobility: TUG test
Voukelatos et al,^42^ 2007	Community dwelling in Central and Southeastern Sydney, Australia; 14% in the tai chi group and 17% in the control group had university education, 43% in the tai chi group and 41% in the control group had intermediate-level education, 16% in the tai chi group and 12% in the control group had below intermediate-level education; remaining participants had secondary education or technical college education	Tai chi (347)	Wait-list control (337)	684	85/69 (7) (total, both groups combined)	83	1/60	16 wk	71	Participants randomized/assessors blinded	Falls: risk of falling (RR), rate of falls (IRR); prospectively monitored daily during 24 wk with a falls calendar that was mailed back to study center monthly; if participants did not send back falls calendars, they were called within 2 wk to assess fall status
Wolf et al,^47^ 1996	Community dwelling; 20.8% in tai chi group and 20.3% in the wellness education group had elementary or high school education, 56.9% in the tai chi group and 51.6% in the wellness education group had college education, and 22.2% in the tai chi group and 28.1% in the wellness education group had graduate school education	Tai chi (72)	Wellness education (64)	137	81/77 (5)	84/75 (4)	2/Minimum of 45 (individual); instructor time, unclear how much time in group setting; participants were asked to perform the exercises 2 times daily for 15 min	15 wk	NR	Participants randomized/assessors blinded	Falls: rate of falls (IRR); monthly calendars or monthly telephone calls by project staff; nurse coordinator verified all fall reports requiring medical attention; upper body strength: HGS
Wolf et al,^43^ 2003	Institutionalized; 80.0% in the tai chi group and 81.6% in the wellness group were White; 80.0% in the tai chi group and 78.0% in the wellness group had high school and beyond education	Tai chi (145)	Wellness education (141)	286	95/81 (7)	94/81 (6)	2/60-90	48 wk	76	Facilities randomized (cluster randomization)/assessors blinded	Falls: risk of falling (RR), rate of falls (IRR); participants reported if they experienced a fall (1) to identify the day and (2) to give details about circumstances and if medical attention was needed; forms were sent to instructor weekly and reviewed by study staff monthly; participants who fell were called to confirm the fall within 1 wk after reviewing of the forms; participants who did not hand in their forms were called for confirmation within 1-2 wk
Woo et al,^44^ 2007	Community dwelling, recruited from community centers in Shatin, Hong Kong; no further statement about socioeconomic status or race/ethnicity	Tai chi (60)	Control (60)	120	50/Men: 68 (2); women: 70 (3)	50/Men: 68 (3); women: 70 (3)	3/NR	12 mo (52 wk)	81	Participants randomized/assessors blinded	Falls: risk of falling (RR); means of assessment NR; balance: OLS (reported average of both legs); upper body strength: HGS (dominant hand, results were combined for men and women before entering the meta-analysis)

Abbreviations: BBS, Berg Balance Scale; FR, functional reach; GP, general practitioner; HGS, handgrip strength; IRR, incidence rate ratio; NR, not reported; OLS, 1-leg stance; RR, risk ratio; SMD, standardized mean difference (Hedges *g*); STS, sit to stand; TAFE, Technical and Further Education; TUG, Timed Up and Go.

^a^ Numbers are rounded to whole numbers.

^b^ Type of dwelling, statements about socioeconomic status (income and education level), and race/ethnicity as reported by the authors.

**Table 2.** Characteristics of Included Trials

Source	Intervention	Adherence, %	Sample size, median (IQR) [range]	Age, weighted mean (SD), y	Intervention duration (follow-up time), median (IQR) [range], wk	Session duration, median (IQR) [range], min
**Risk of falling (RR) (n = 8 trials including 1579 participants)**^a^						
Choi et al,^37^ 2004	Tai chi with music	70	127 (73.25-212.50) [30-684]	73.19 (4.94)	22 (15-30) [8-48]	60 (42.4-60) [35-60]^b^
Eyigor et al,^38^ 2009	Turkish folk dance	Not reported				
Huang et al,^39^ 2010	Tai chi	Not reported				
Li et al,^40^ 2005	Tai chi	61 of 72 lessons scheduled				
Trombetti et al,^41^ 2011	Dalcroze eurhythmics	83				
Voukelatos et al,^42^ 2007	Tai chi	71				
Wolf et al,^43^ 2003	Tai chi	76				
Woo et al,^44^ 2007	Tai chi	81				
**Rate of falls (IRR) (n = 7 trials including 2012 participants)**^a^						
Chyu et al,^45^ 2010	Tai chi	94	188 (135-408) [54-702]	74.40 (4.33)^c^	24 (20-36) [15-52]	60 (60-60) [45-70]
Li et al,^40^ 2005	Tai chi	61 of 72 lessons scheduled				
Merom et al,^46^ 2016	Folk or ballroom dance	51				
Trombetti et al,^41^ 2011	Dalcroze eurhythmics	83				
Voukelatos et al,^42^ 2007	Tai chi	71				
Wolf et al,^47^ 1996	Tai chi	Not reported				
Wolf et al^43^ 2003	Tai chi	76				
**Balance (SMD) (n = 15 trials including 1476 participants)**^a^						
Alves et al,^48^ 2013	Ballroom dance	90	77 (51-125.5) [23-269]	72.90 (4.2)	16 (21-24) [8-48]	60 (50-60) [35-120]^b^
Bennett et al,^49^ 2018	Line dancing	80				
Choi et al,^37^ 2004	Tai chi with music	70				
Eyigor et al,^38^ 2009	Turkish folk dance	Not reported				
Hopkins et al,^53^ 1990	Aerobic dance	Not reported				
Huang et al,^39^ 2010	Tai chi	Not reported				
Li et al,^40^ 2005	Tai chi	85 (61 of 72 lessons scheduled)				
Li et al,^51^ 2008	Tai chi	92				
Logghe et al,^52^ 2009	Tai chi	47% Of participants attended at least 80% of classes				
Pereira et al,^53^ 2008	Tai chi	Not reported				
Serano-Guzmann et al,^54^ 2016	Flamenco and sevillanas	100				
Sun et al,^55^ 2015	Tai chi	Not reported				
Taylor-Piliae et al,^56^ 2010	Tai chi	77				
Trombetti et al,^41^ 2011	Dalcroze eurhythmics	83				
Woo et al,^44^ 2007	Tai chi	81				
**Mobility (SMD) (n = 13 trials including 1379 participants)**^a^						
Alves et al,^48^ 2013	Ballroom dance	90	54 (50-97) [19-451]	73.02 (3.25)	16 (12-24) [8-52]	60 (40-60) [40-120]
Cepeda et al,^57^ 2015	Ballroom dance	91				
Chyu et al,^45^ 2010	Tai chi	94				
Cruz-Ferreira et al,^58^ 2015	Creative dance	85				
Frye et al,^59^ 2007	Tai chi	91.4% Of participants attended at least 80% of classes				
Hopkins et al,^53^ 1990	Low-impact aerobic dance	Not reported				
Huang et al,^39^ 2010	Tai chi	Not reported				
Hui et al,^60^ 2009	Low-impact aerobic dance	91.3				
Li et al,^40^ 2005	Tai chi	85 (61 of 72 lessons scheduled)				
Noradechanunt et al,^61^ 2017	Tai chi	85				
Serano-Guzmann et al,^54^ 2016	Flamenco and sevillanas	100				
Taylor et al,^62^ 2012	Tai chi	1 Time per week: 79; 2 times per week: 72				
Trombetti et al,^41^ 2011	Dalcroze eurhythmics	83				
**Lower body strength (SMD) (n = 13 trials including 1613 participants)**^a^						
Chyu et al,^45^ 2010	Tai chi	94	54 (38-97) [19-530]	73.14 (3.30)^c^	12 (12-24) [6-52]	60 (50-60) [40-120]
Cruz-Ferreira et al,^58^ 2015	Creative dance	85				
Eyigor et al,^38^ 2009	Turkish folk dance	Not reported				
Frye et al,^59^ 2007	Tai chi	91.4% Of participants attended at least 80% of classes				
Hopkins et al,^53^ 1990	Low-impact aerobic dance	Not reported				
Hui et al,^60^ 2009	Low-impact aerobic dance	91.3				
Janyacharoen et al,^53^ 2013	Thai dance	Not reported				
McKinley et al,^63^ 2008	Adapted tango	>90				
Merom et al,^46^ 2016	Folk or ballroom dance	51				
Merom et al,^65^ 2016	Ballroom dance	66				
Noradechanunt et al,^61^ 2017	Tai chi	85				
Taylor et al,^62^ 2012	Tai chi	1 Time per week: 79; 2 times per week: 72				
Taylor-Piliae et al,^56^ 2010	Tai chi	77				
**Upper body strength (SMD) (n = 4 trials including 414 participants)**^a^						
Frye et al,^59^ 2007	Tai chi	91.4% Of participants attended at least 80% of classes	116 (95-124.5) [44-138]	70.78 (3.3)	20.5 (14.25-32.5) [12-52]	60 (52.5-60) [45-60]^b^
Sun et al,^55^ 2015	Tai chi	Not reported				
Wolf et al,^47^ 1996	Tai chi	Not reported				
Woo et al,^44^ 2007	Tai chi	81				
**Total**						
All 29 included trials	NA	NA	77 (44-136) [19-702]	73.10 (4.22)^c^	16 (12-24) [6-52]	60 (50-60) [35-120]^b^

Abbreviations: IQR, interquartile range; IRR, incidence rate ratio; NA, not applicable; RR, risk ratio; SMD, standardized mean difference (Hedges *g*).

^a^ Total sum of participants of all trials included within specific outcome.

^b^ Woo et al^44^ do not report on session duration; therefore, numbers were calculated without this study.

^c^ For Merom et al,^46^ see Table 1 for reported details. Mean age was calculated as follows: intervention group: (275 × 0.57 × 70 + 275 × 0.43 × 85)/275 = 76.45; control group: 247 × 0.65 × 70 + 247 × 0.35 × 85)/247 = 75.25; and estimated SD = 8.0 for both the intervention and control groups.

Overall, the sample size of the trials varied greatly, from 19 up to 684 participants. Most trials^38,39,40,41,42,44,45,47,48,49,50,51,52,53,54,55,56,57,58,59,60,61,62,63,64,65^ included community-dwelling older adults, with most participants being women. Seven trials^37,40,41,43,47,52,64^ reported the mean age of their population being at least 70 years. Three trials^37,43,46^ were conducted among participants living in independent living facilities. Five trials^37,39,43,46,60^ used cluster randomization.

Within the 29 trials, the interventions lasted between 6 weeks and 12 months, and the duration of intervention sessions ranged from 35 to 120 minutes. The frequency of the study intervention varied between once per week^41,42^ and 4 times per week,^51^ with most trials reporting 2 times per week^43,46,47,48,49,52,55,56,60,61,62,64,65^ or 3 times per week.^37,38,39,40,44,45,50,53,54,57,58,59,63^ Adherence was at least 80% in 15 of the 22 trials^40,41,44,45,48,49,51,52,54,57,58,59,60,61,64^ that reported adherence.

In terms of type of dance-based mind-motor activity, 13 trials^38,41,46,48,49,50,54,57,58,60,63,64,65^ investigated activities that involved dance styles, such as ballroom or folk dances, and 16 trials^37,39,40,42,43,44,45,47,51,52,53,55,56,59,61,62^ investigated tai chi.

More than half of the trials were conducted in North America^40,43,45,47,48,49,50,56,59,64^ (10 trials) or Asia^37,44,51,55,60,63^ (7 trials). Five trials each were from Europe^38,41,52,54,58^ and Oceania,^42,46,61,62,65^ and 2 trials^53,57^ were from South America.

### Primary Outcomes: Risk of Falling and Rate of Falls

**Figure 1.**
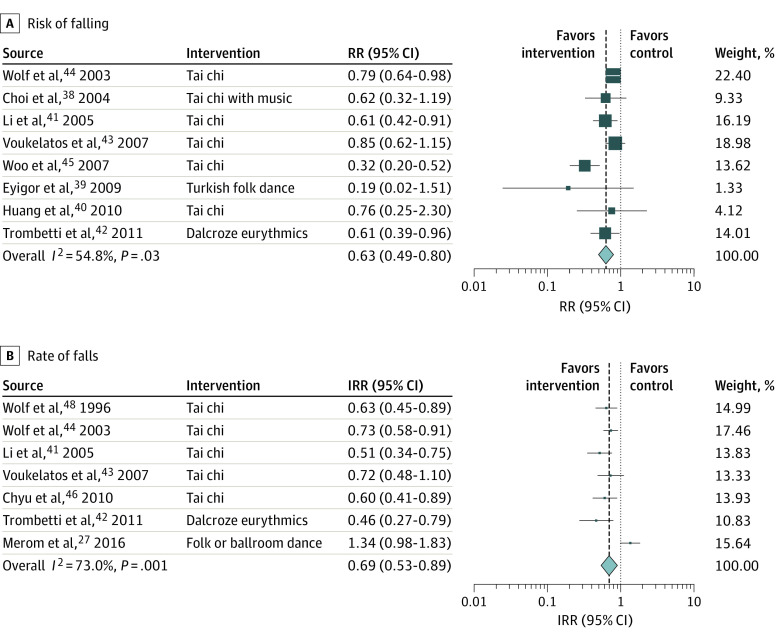
Association of Dance-Based Mind-Motor Activities With Falls For risk of falling, the total sample size by pooling 8 trials was 1579. For rate of falls, the total sample size by pooling 7 trials was 2012. Weights are from random-effects analysis. Box sizes correspond to precision; the bigger the box, the more precise. Precision was determined as the inverse of the variance. IRR indicates incidence rate ratio; RR, risk ratio.

Dance-based mind-motor activities were associated with 37% reduction in risk of falling (RR, 0.63; 95% CI, 0.49-0.80) (Figure 1A) based on 8 trials^37,38,39,40,41,42,43,44^ of 1579 participants, with a weighted mean (SD) age of 73.2 (4.9) years, a median sample size of 127 (range, 30-684), and median intervention duration of 22 weeks (range, 8-48 weeks).

Prespecified subgroup analyses revealed a significant association with reduction of risk of falling for trials that conducted interventions 3 times per week or more (RR, 0.47; 95% CI, 0.31-0.72) and for trials with durations between 12 and 24 weeks (RR, 0.71; 95% CI, 0.58-0.86 (eAppendix in the Supplement).

Two sensitivity analyses were performed for risk of falling, one excluding the trial by Woo et al,^44^ which was driving the pooled result, and one excluding the trial by Eyigor et al,^38^ from which unpublished results were taken. For both sensitivity analyses, the pooled RR remained statistically significant in favor of the intervention groups (RR, 0.74; 95% CI, 0.64-0.86 for the analysis excluding the trial by Woo et al,^44^ and RR, 0.64; 95% CI, 0.50-0.82 for the analysis excluding the trial by Eyigor et al^38^) (eAppendix in the Supplement).

For rate of falls, dance-based mind-motor activities were associated with a reduction by 31% (IRR, 0.69; 95% CI, 0.53-0.89; 7 trials) (Figure 1B) based on 7 trials of 2012 participants with a weighted mean (SD) age of 74.4 (4.3) years, a median sample size of 188 (range, 54-684), and a median intervention duration of 24 weeks (range, 15-52 weeks).

Subgroup analyses suggested this association was most pronounced in trials that conducted the intervention 3 times per week or more (IRR, 0.55; 95% CI, 0.42-0.73) and for trials that lasted between 12 and 24 weeks (IRR, 0.59; 95% CI, 0.49-0.71) (eAppendix in the Supplement).

### Secondary Outcome: Physical Function

**Figure 2.**
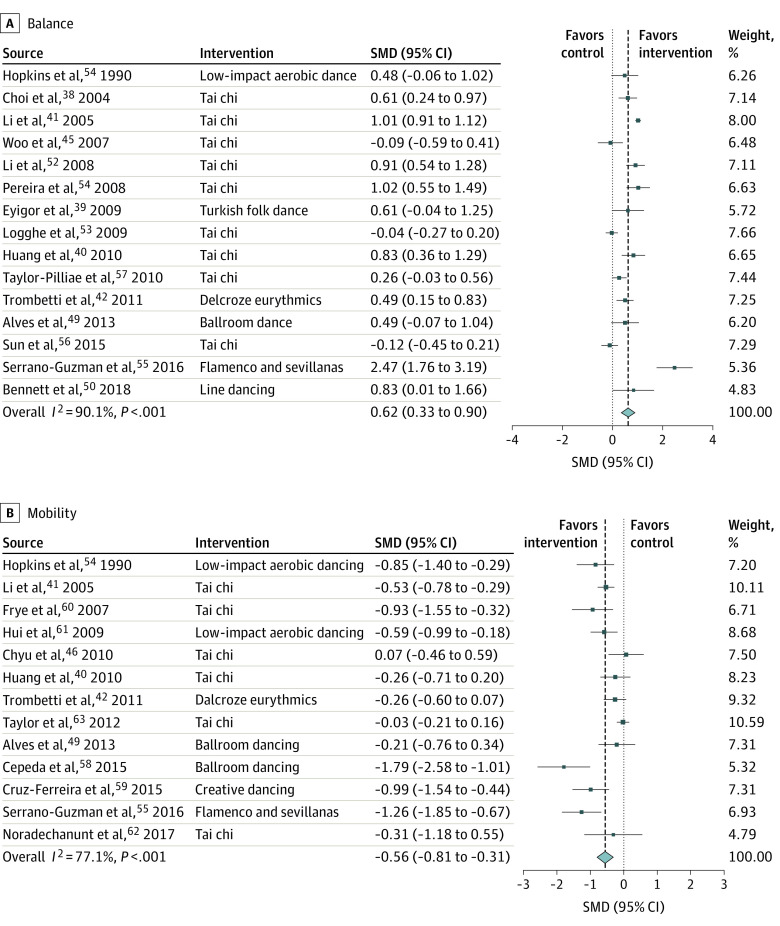
Association of Dance-Based Mind-Motor Activities With Balance and Mobility Effect sizes are Hedges *g* standardized mean differences (SMDs). Weights are from random-effects analysis. Box sizes correspond to precision; the bigger the box, the more precise. Precision was determined as the inverse of the variance for each estimate. For balance (A), assessments included the Berg Balance Scale, 1-leg stance, and functional reach. For the Berg Balance Scale and 1-leg stance, an increase of time indicates an improvement in the test result. For functional reach, an increase in reached distance indicates an improvement. Hence, positive values would favor the intervention groups over the control groups. For mobility (B), all included trials assessed mobility with the Timed Up and Go test. Decreased completion time indicated improvement in the Time Up and Go Test result. Hence, negative values would favor the intervention groups over the control groups.

An association was found between dance-based mind-motor activities and improved balance (SMD, 0.62; 95% CI, 0.33-0.90) (Figure 2A) in 15 trials^37,38,39,40,41,44,48,49,50,51,52,53,54,55,56^ of 1476 participants, with a weighted mean (SD) age of 72.3 (4.3) years, a median sample size of 77 (range, 23-269), and a median intervention duration of 16 weeks (range, 8-48 weeks). Subgroup analyses by intervention type found a higher SMD for non–tai chi (SMD, 0.86; 95% CI, 0.33-1.39) activities. Interventions performed 3 times per week or more were associated with a higher SMD (SMD, 0.84; 95% CI, 0.54, 1.14). Sensitivity analyses excluding the trial by Hopkins et al^50^ (published much earlier than the others) or the trial by Serrano-Guzman et al^54^ (driving the result) also found an association between dance-based mind-motor and improved balance (SMD, 0.63; 95% CI, 0.33-0.93 for the analysis excluding the trial by Hopkins et al^50^ and SMD, 0.51; 95% CI, 0.24-0.78 for the analysis excluding the trial by Serrano-Guzman et al^54^).

An association was also found between dance-based mind-motor activities and improved mobility (SMD, –0.56; 95% CI, –0.81 to –0.31) (Figure 2B) based on 13 trials^39,40,41,45,48,50,54,57,58,59,60,61,62^ of 1379 participants, with a weighted mean (SD) age of 73.0 (3.3 years), a median sample size of 54 (range, 19-451), and a median intervention duration of 16 weeks (range, 8-52 weeks). For mobility, decreased completion time indicated improvement in Timed Up and Go test results. Hence, negative values would favor the intervention groups over control groups. Subgroup analyses found positive associations for non–tai chi activities (SMD, –0.79; 95% CI, –1.16 to –0.42) and for activities with 3 sessions or more per week (SMD, –0.76; 95% CI, –1.10 to 0.42). The results remained positively associated within sensitivity analyses that excluded the trial by Hopkins et al^50^ or the trial driving the results by Cepeda et al^57^ (SMD, –0.54; 95% CI, –0.80 to –0.28 for the analysis excluding the trial by Hopkins et al^50^ and SMD, –0.48; 95% CI, –0.71 to –0.25 for the analysis excluding the trial by Cepeda et al^57^).

**Figure 3.**
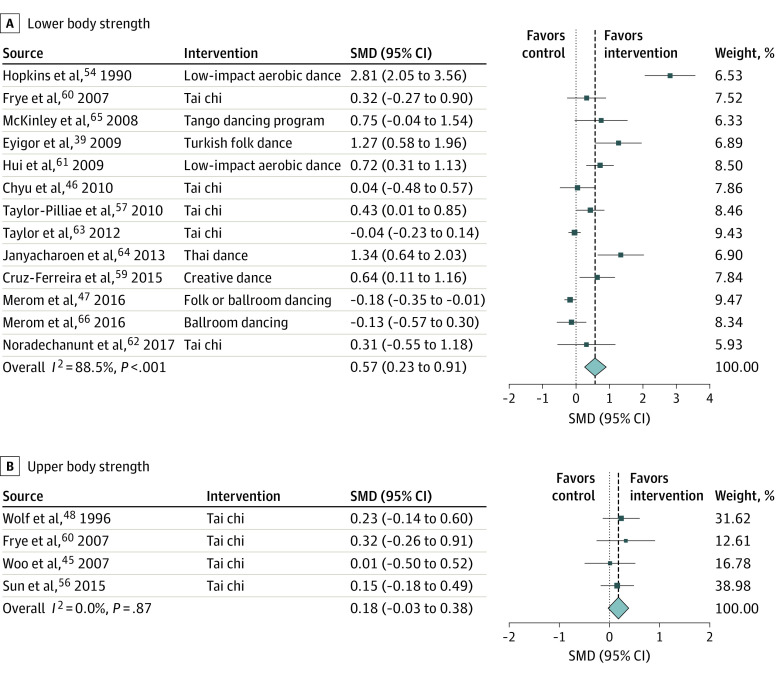
Association of Dance-Based Mind-Motor Activities With Body Strength Effect sizes are Hedges *g* standardized mean differences (SMDs). Weights are from random-effects analysis. Box sizes correspond to precision; the bigger the box, the more precise. Precision was determined as the inverse of the variance for each estimate. For lower body strength (A), assessments included the 5 times sit to stand (STS) test (the time to complete 5 stands is measured) and the 30-second STS test (number of stands completed within 30 seconds is measured). Effect sizes of the 5 times STS test were multiplied by −1 to achieve same direction signaling improvement as the effect sizes of the 30-second STS test. Hence, positive values would favor the intervention groups over the control groups. For upper body strength (B), all included trials assessed UBS using handgrip strength. Increasing values indicate an improvement in the test result. Hence, positive values would favor the intervention groups over the control groups.

Dance-based mind-motor activities were associated with improved lower body strength (SMD, 0.57; 95% CI, 0.23-0.91) (Figure 3A), based on 13 trials^38,45,50,56,58,59,60,61,62,63,64,65^ with 1613 participants with a weighted mean (SD) age of 73.1 (3.3) years, a median sample size of 54 (range, 19-530), and a median intervention duration of 12 weeks (range, 6-52 weeks). Subgroup analyses supported a positive association with improved lower body strength for non–tai chi activities (SMD, 0.86; 95% CI, 0.25-1.47) and intervention frequencies of 3 times per week or more (SMD, 1.04; 95% CI, 0.31-1.77). In a sensitivity analysis excluding the early trial by Hopkins et al,^50^ the SMD remained significant (SMD, 0.39; 95% CI, 0.12-0.65). No significant association was found between dance-based mind-motor activities and upper body strength (SMD, 0.18; 95% CI, −0.03 to 0.38) (Figure 3B) based on 4 trials^44,47,55,59^ with 414 participants. For subgroup and sensitivity analyses of physical function outcomes, see the eAppendix in the Supplement.

### Assessment of Small-Study Effects

The limited number of trials did not allow for the assessment of small-study effects for the risk of falling, rate of falls, and upper body strength.^22^ For balance, visual inspection of the funnel plot suggested asymmetry, but there was no evidence of small-study effects based on the Egger test (intercept, −1.59; SE, 1.45; *P* = .29). For mobility and lower body strength, the funnel plots showed asymmetry, and the Egger test result was statistically significant (intercept, −2.87; SE, 1.04; *P* = .02 for mobility and intercept, 4.04; SE, 1.05; *P* = .003 for lower body strength), suggesting that small-study effects cannot be ruled out.

### Assessment of Heterogeneity

Moderate heterogeneity was found for the primary outcomes (risk of falling: *I*^2^ = 54.8%, *P* = .03; rate of falls: *I*^2^ = 73.0%, *P* = .001). For secondary outcomes, substantial heterogeneity was found in all domains except upper body strength (balance: *I*^2^ = 90.1%, *P* < .001; mobility: *I*^2^ = 77.1%, *P* < .001; lower body strength: *I*^2^ = 88.5%, *P* < .001; upper body strength: *I*^2^ = 0.0%, *P* = .87).

### Assessment of Bias

Based on the Cochrane tool of bias,^66^ a high risk of bias was found for at least 1 domain in 10 trials^37,38,39,46,49,51,58,59,60,65^ and an unclear risk of bias in at least 1 domain for every included trial (eAppendix in the Supplement).^66^

## Discussion

In this systematic review and meta-analysis, which included 29 trials of 4239 healthy older adults, there was a significant association between dance-based mind-motor activities, including both tai chi and non–tai chi activities, and reductions in the risk of falling and the rate of falls. The association of dance-based mind-motor activities with consistent improvements in balance, mobility, and lower body strength supports these findings. Notably, the predefined subgroup analyses suggest that greater frequency (≥3 times per week) and a duration of the intervention between 12 and 24 weeks are associated with greater benefits from these interventions with regard to fall and functional outcomes.

For fall prevention, the analysis of dance-based mind-motor interventions are in alignment with findings of prior meta-analyses^67,68^ of multicomponent physical exercise interventions or tai chi alone. Although the analyses for the primary outcomes included more trials that investigated tai chi than non–tai chi dance-based mind-motor activities, the results by subgroups of type of dance-based mind-motor activities suggest that non–tai chi trials might have similar associations with risk of falls and fall rate reductions. In particular, there was a consistently stronger association with improvement of function and non–tai chi dance-based mind-motor interventions for balance, mobility, and lower body strength, and the results therefore extend and strengthen the evidence base of exercise trials that focus on multitasking skills for fall prevention among healthy older adults.

To our knowledge, this meta-analysis is the first that aims to summarize the associations of dance-based mind-motor activities beyond tai chi with the risk of falling and the rate of falls among healthy older adults. Earlier systematic reviews that investigated dance-based mind-motor activities among healthy older adults suggested a beneficial effect for fall prevention solely based on the improvement of balance or strength^9,10,11,69^ or included tai chi interventions only.^68^ Other meta-analyses on the effect of dance-based mind-motor activities preselected on cardiovascular risk,^70^ Parkinson disease,^71^ or cognitive^12,72^ function. Sheldon et al^67^ report subgroup analyses only for 1 trial in the category dance, whereas the present study additionally included 2 trials that investigated non–tai chi activities for each outcome (risk of falling^38,41^ and rate of falls^41,46^).

Furthermore, the present meta-analysis supports benefits of dance-based mind-motor activities for several dimensions of physical function, including balance, mobility, and lower body strength. The observed nonsignificant association on upper body strength may be explained by the fact that the upper extremities in dance-based mind-motor activities are used for expression and partnering rather than building strength or body weight support.

### Strengths and Limitations

This study has strengths and limitations. Strengths include the comprehensive search strategy within 10 different databases, which incorporated unpublished information from authors^38^ and was built on the conceptual framework established by the Prevention of Falls Network Europe (ProFaNE).^67^ In addition to ProFaNE’s definition of 3-dimensional training, this study combined mind and motor abilities, as well as the domain of social interaction. In this meta-analysis, 13 of 29 trials^38,41,46,48,49,50,54,57,58,60,63,64,65^ tested dance styles.

This study found consistent associations between dance-based mind-motor activities and improvement for falls and physical function, and the associations remained significant for all sensitivity analyses performed with influential studies excluded. Finally, relevant to implementation of dance-based mind-motor activities into public health strategies, most trials included in this meta-analysis reported at least 80% adherence, which may be better than previously reported adherence rates for other structured exercise interventions.^25,67^

This meta-analysis also has limitations. Because of the limited number of non–tai chi trials, several of these trials had to be excluded, giving a stronger weight to the tai chi interventions. However, subgroup analyses excluding the tai chi trials support a similar positive association of non–tai chi activities with balance, mobility, and lower body strength as tai chi. Although data for risk of falling and fall rate were collected with sufficient quality, aspects of physical function, such as balance and strength, were evaluated in part with nonstandardized and noncomparable assessment devices, such as balance platforms or isokinetic machines, which led to the exclusion of several trials that reported on physical function. Finally, these results might not be generalizable to older men because all but 1 trial included mainly women.^39,44,51^

## Conclusions

Although these results found significant positive associations across risk of falling, rate of falls, and 3 of 4 investigated functional measures and are therefore promising in their consistency and effect size for fall prevention, the study also documented limitations in the quality of individual trials. This is true especially for the non–tai chi interventions. Tai chi is among the best-studied activities for older adults.^68^ However, additional high-quality trials investigating other types of dance-based mind-motor activities are needed to evaluate options for populations who do not have a tradition of tai chi practice but do have strong cultural bonds to activities such as folk and ballroom dancing or eurhythmics. Additional trials are needed to investigate dance-based mind-motor activities, considering optimal duration and frequency for most effective fall prevention among healthy older adults.

## Notes

Supplement. **eAppendix. **Supplementary Material
