# Errors in Citations, Data Reporting, and the Supplement

**DOI:** 10.1001/jamanetworkopen.2020.37105

**Published:** 2021-01-22

In the Original Investigation titled “Association of Dance-Based Mind-Motor Activities With Falls and Physical Function Among Healthy Older Adults: A Systematic Review and Meta-analysis,”^1^ published online September 25, 2020, there were errors in the References, data presentation, affiliations, and Supplement. In a number of places in the Results section, tables, and figures, reference citations were misnumbered, misplaced, or missing, and the publication date of 1 study, by Choi et al,^2^ should have read 2005. The name of the author of a study^3^ should have read Sherrington. In the second paragraph of the Results section, the 7 trials reported as having study populations with a mean age of at least 70 years had study populations with a mean age of at least 75 years. Also in Results, in the third paragraph of the section titled “Secondary Outcome: Physical Function,” the results for the association between improved lower body strength and non–tai chi activities should have read SMD, 0.86; 95% CI, 0.25 to 1.46. In Table 2, the range of numbers of patients in trials showing the rate of falls should have read 54 to 684; also in Table 2, the age (weighted mean) in years of patients in studies including balance should have read 72.90 (4.3). One affiliation should have read Center on Aging and Mobility, University Hospital Zurich, City Hospital Waid and Triemli and University of Zurich, Zurich, Switzerland. In addition, an older version of the Supplement was inadvertently published and has been replaced with the updated version. This article has been corrected.^1^
