# Supplementary material for: Association of Dance-Based Mind-Motor Activities With Falls and Physical Function Among Healthy Older Adults: A Systematic Review and Meta-analysis
Source: JAMA Netw Open. 2020 Sep 25;3(9):e2017688. doi: 10.1001/jamanetworkopen.2020.17688 (PMC7519422; doi:10.1001/jamanetworkopen.2020.17688)
Supplement: Supplement. — eAppendix. Supplementary Material [file jamanetwopen-e2017688-s001.pdf]

Supplementary Online Content

Mattle M, Chocano-Bedoya PO, Fischbacher M, et al. Association of dance-based mind-motor activities with falls and physical function among healthy older adults: a systematic review and meta-analysis. *JAMA Netw Open*. 2020;3(9):e2017688.
doi:10.1001/jamanetworkopen.2020.17688

eAppendix. Supplementary Material

This supplementary material has been provided by the authors to give readers additional information about their work.

© 2020 Mattle M et al. *JAMA Network Open.*

Search Syntax in PubMed

("Aged"[MeSH] OR older[Title/Abstract] OR
elderly[Title/Abstract] OR senior*[Title/Abstract] OR old-age*[Title/Abstract] OR aged[Title/Abstract])
AND
("Dance Therapy"[MeSH] OR "Dancing"[MeSH] OR "Exercise Movement Techniques"[MeSH] OR
dance* [Title/Abstract] OR dancing[Title/Abstract] OR ballet*[Title/Abstract] OR jazz*[Title/Abstract]
OR hiphop[Title/Abstract] OR salsa*[Title/Abstract] OR zumba*[Title/Abstract] OR
aerobic*[Title/Abstract] OR taiji[Title/Abstract] OR taijiquan[Title/Abstract] OR
"tai chi"[Title/Abstract] OR eurythm*[Title/Abstract])
AND
("Accidental Falls"[MeSH] OR
Falls[Title/Abstract] OR faller* [Title/Abstract] OR falling[Title/Abstract] OR slip*[Title/Abstract] OR
stumble*[Title/Abstract] OR tumble* [Title/Abstract] OR fell[Title/Abstract] OR
balance[Title/Abstract] OR strength[Title/Abstract] OR mobility[Title/Abstract] OR
flexibility[Title/Abstract])

© 2020 Mattle M et al. *JAMA Network Open.*

Inclusion flow

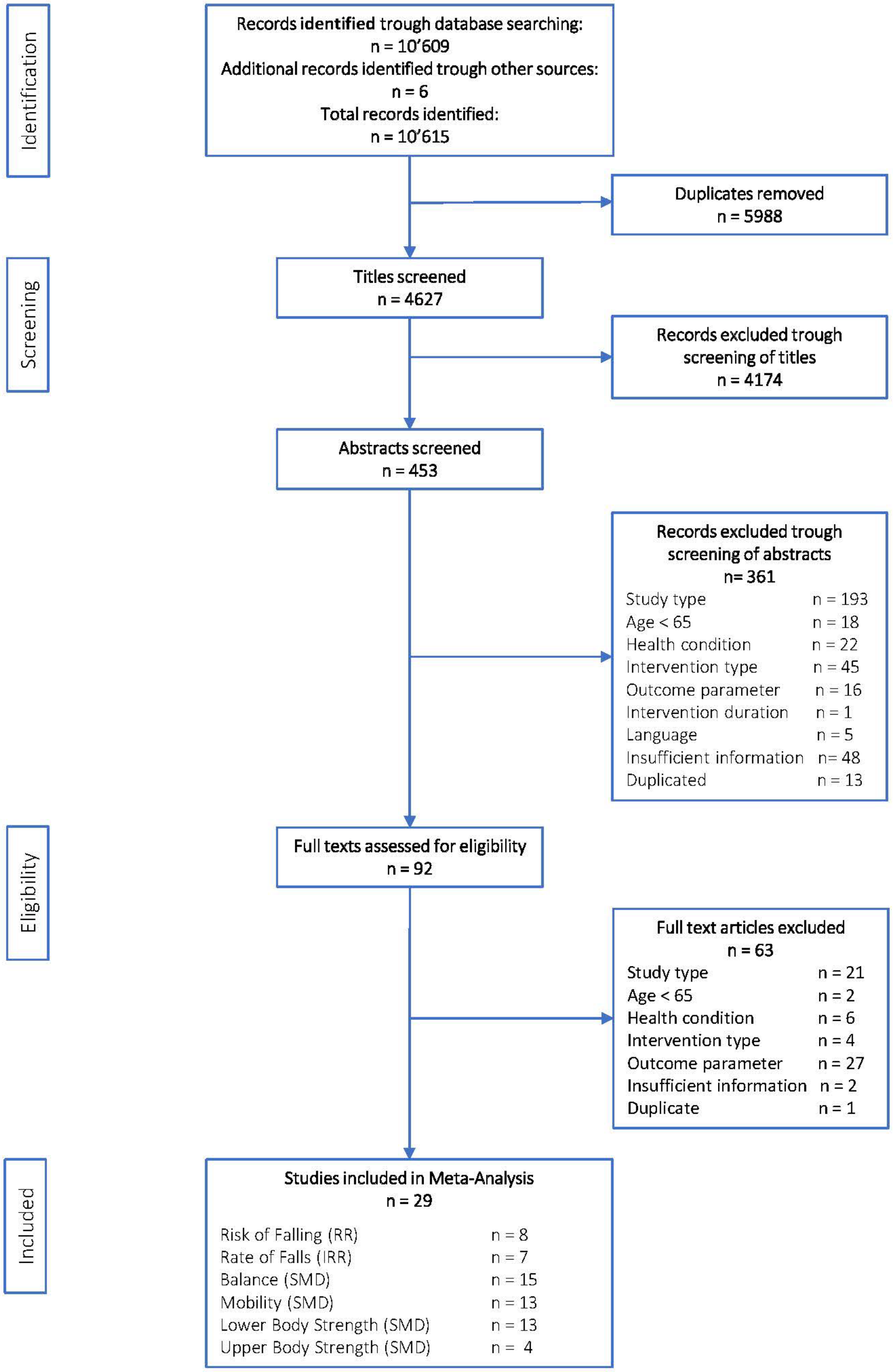

© 2020 Mattle M et al. *JAMA Network Open.*

Subgroup Analyses Risk of Falling

Risk of Falling, total N=8 trials	Risk Ratio (95%CI)	I^2
**Full**	**0.63 (0.49, 0.80)**	**54.8 %**
**by intervention type**		
Tai Chi	0.64 (0.48, 0.85)	63.5 %
Non-Tai Chi	0.53 (0.26, 1.12)	15.5 %
**by intervention frequency**		
< 3 times / week	0.78 (0.66, 0.92)	0.0%
≥ 3 times / week	0.48 (0.31, 0.72)	47.1 %
**by intervention duration**		
> 24 weeks	0.52 (0.21, 1.25)	91.4 %
12 - 24 weeks	0.71 (0.58, 0.86)	0.0%
< 12 weeks	0.19 (0.02, 1.51)	--
**by type of dwelling**		
community dwelling	0.58 (0.41, 0.82)	60.9 %
living facilities	0.77 (0.63, 0.95)	0.0%
**by type of randomization**		
participants randomized	0.56 (0.38, 0.82)	68.4 %
cluster randomized	0.77 (0.63, 0.94)	0.0%
**by duration of one class**		
< 60 min	0.77 (0.63, 0.94)	0.0%
≥ 60 min	0.69 (0.54, 0.89)	19.6 %
Woo et al. do not report time		
**by total contact time**		
< 90 min	0.73 (0.61, 0.87)	0.0%
≥ 90 min	0.75 (0.55, 1.02)	- 25.7 %
Woo et al. do not report duration of classes		

© 2020 Mattle M et al. *JAMA Network Open.*

Assciation of dance-based mind-motor activities with risk of falling by intervention type

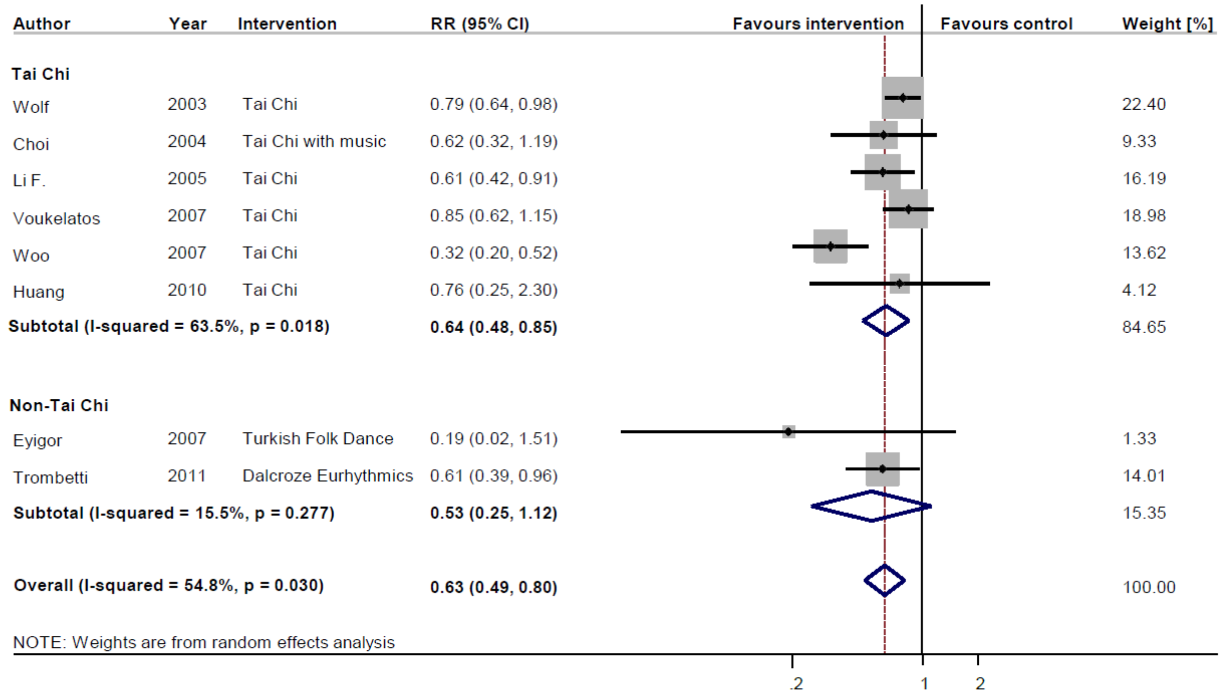

Association of dance-based mind-motor activities with risk of falling by intervention frequency

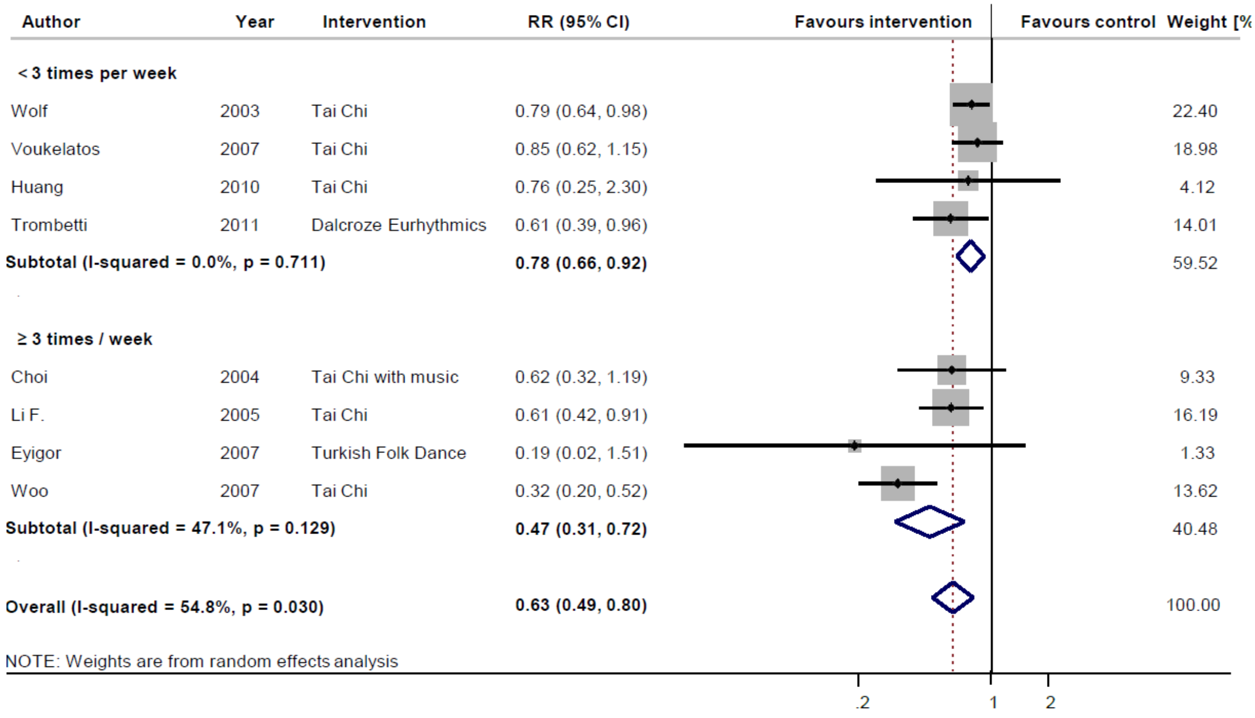

© 2020 Mattle M et al. *JAMA Network Open.*

Association of dance-based mind-motor activities with risk of falling by intervention duration

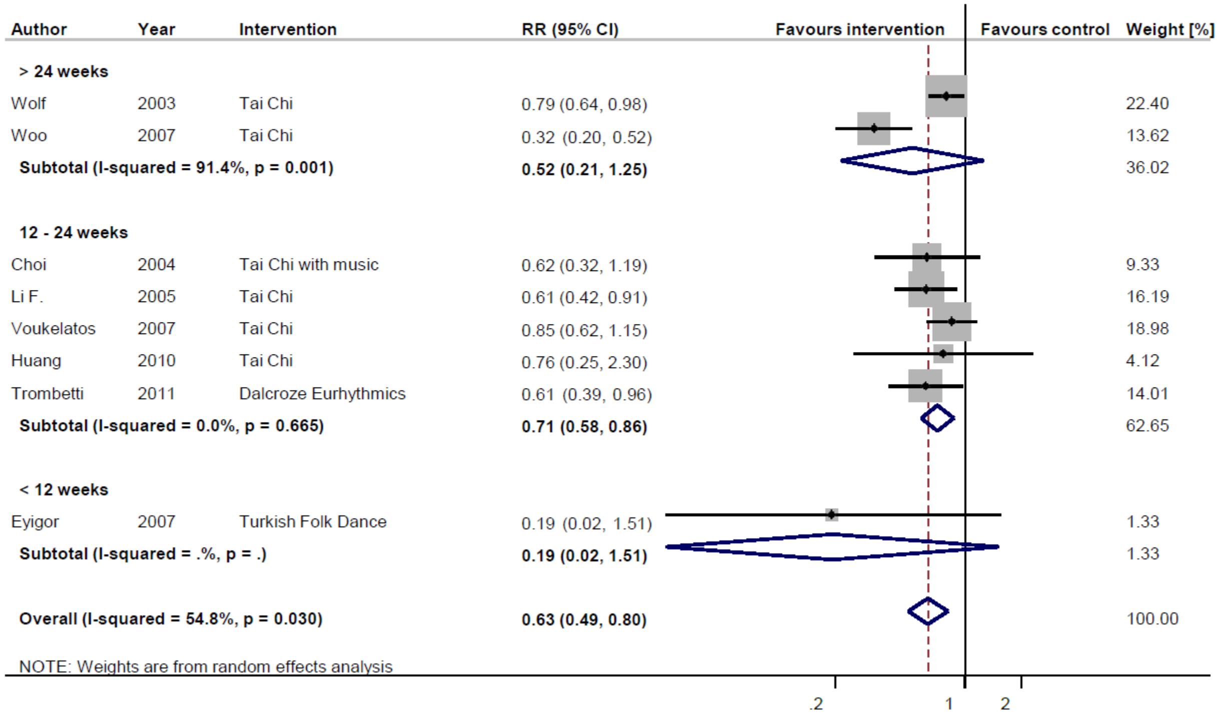

Association of dance-based mind-motor activities with risk of falling by type of dwelling

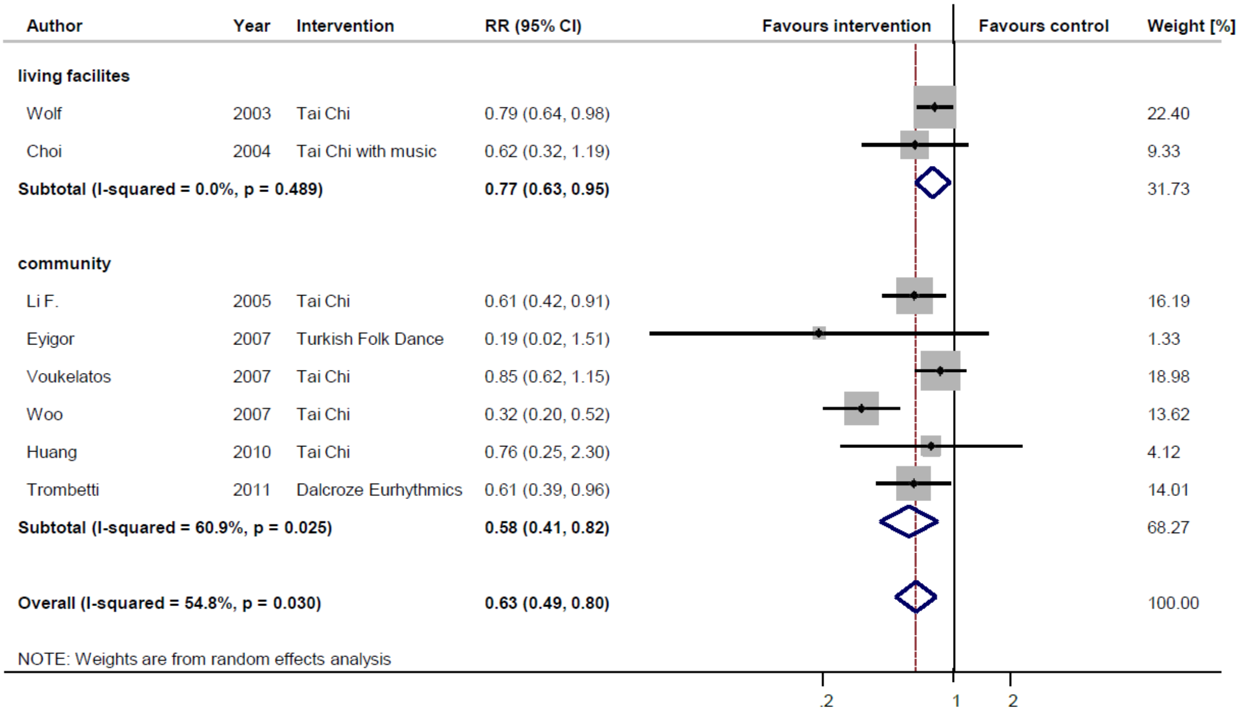

© 2020 Mattle M et al. *JAMA Network Open.*

Association of dance-based mind-motor activities with risk of falling by type of randomization

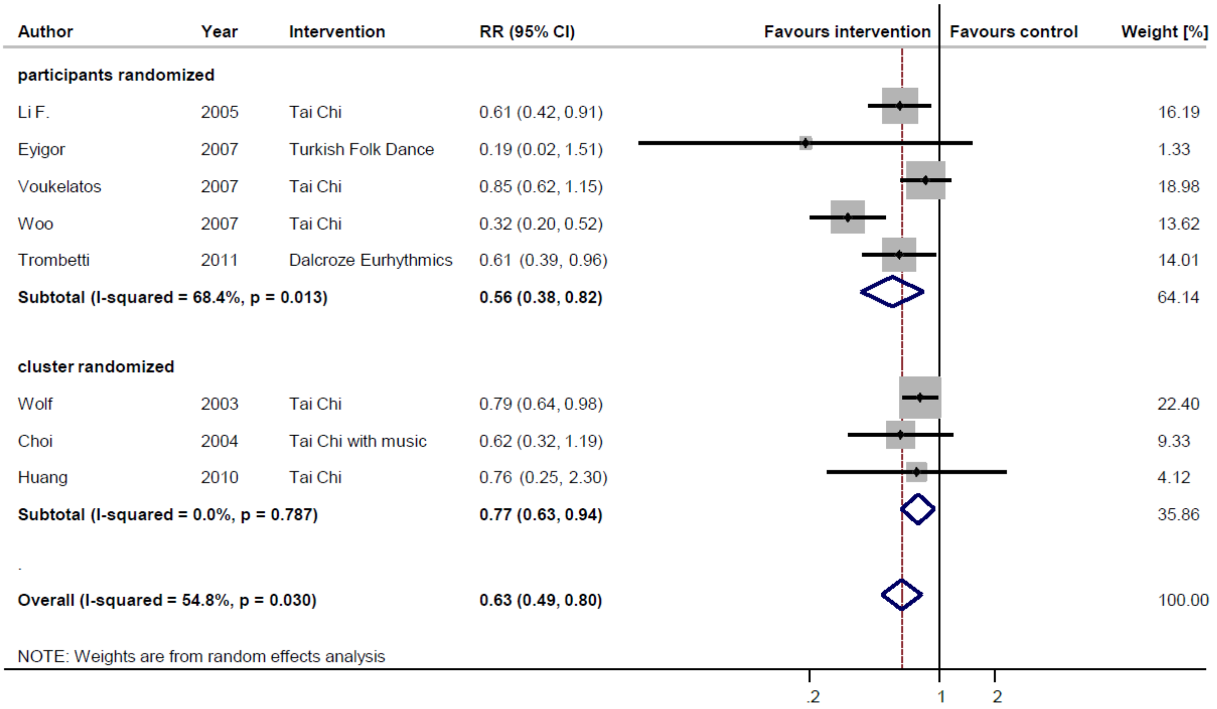

Association of dance-based mind-motor activities with risk of falling by duration of one session

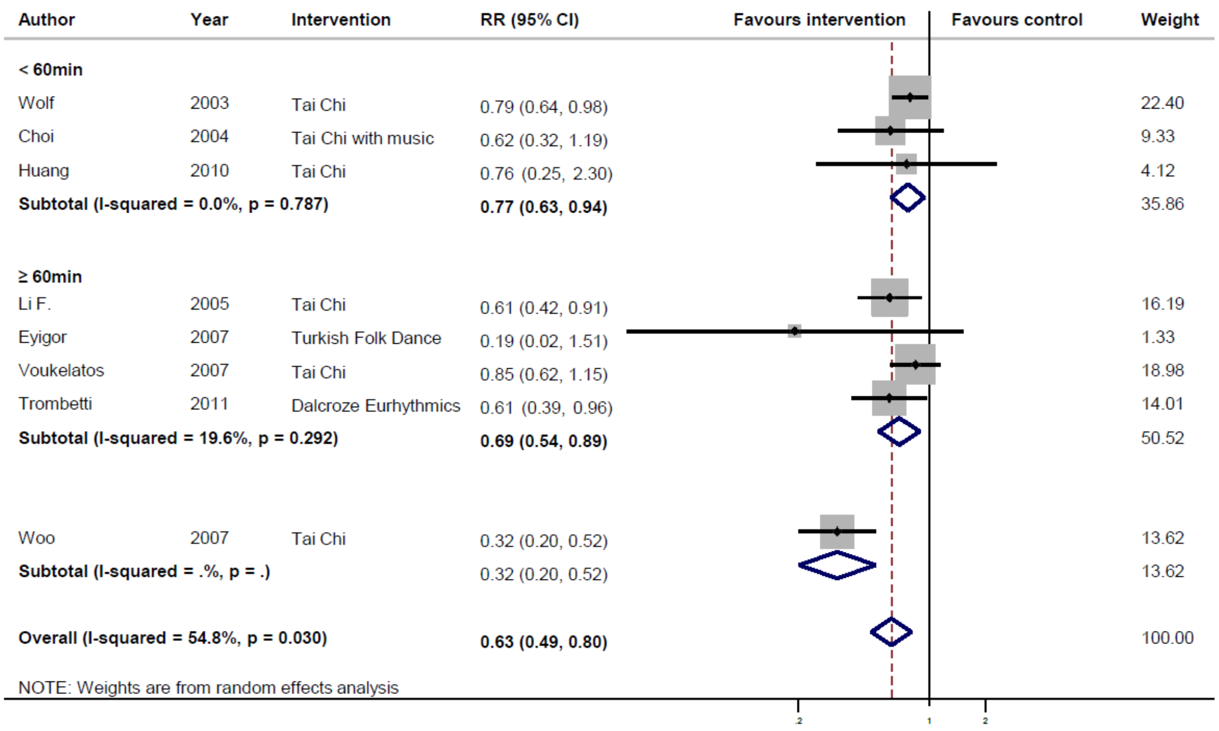

© 2020 Mattle M et al. *JAMA Network Open.*

Association of dance-based mind-motor activities with risk of falling by total contact time

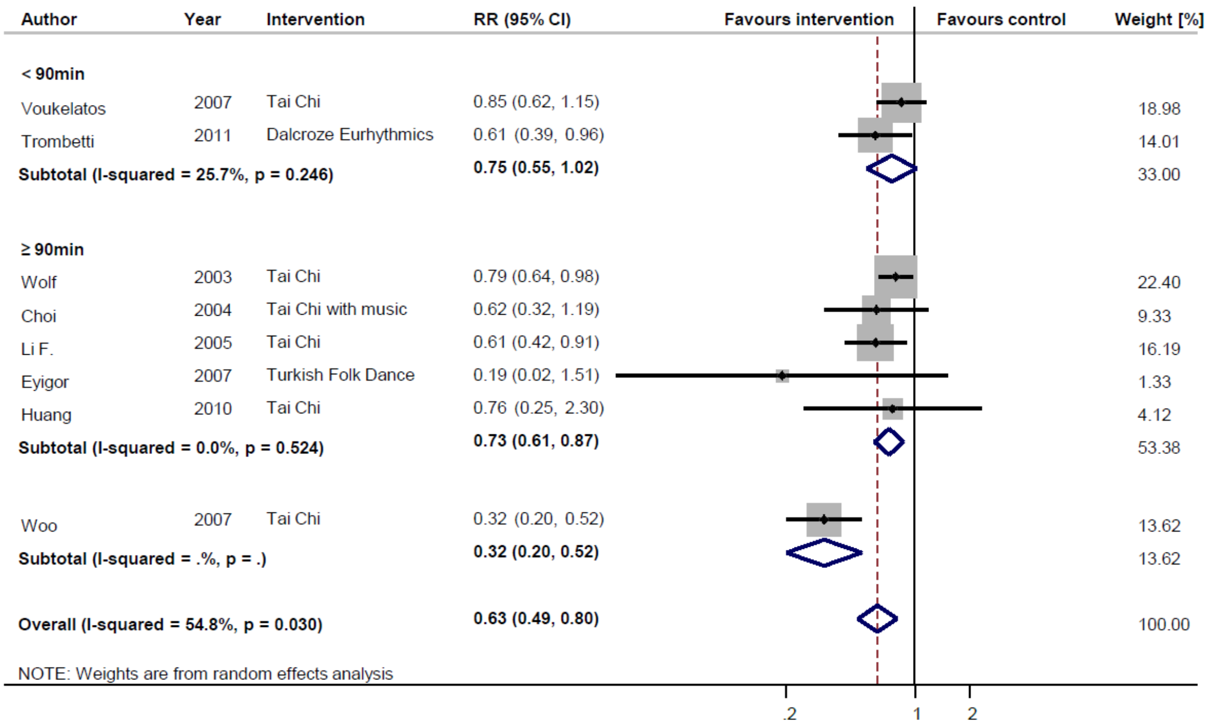

© 2020 Mattle M et al. *JAMA Network Open.*

Sensitivity Analyses Risk of Falling

Association of dance-based mind-motor activities with risk of falling Sensitivity analysis 1: without Woo et al.

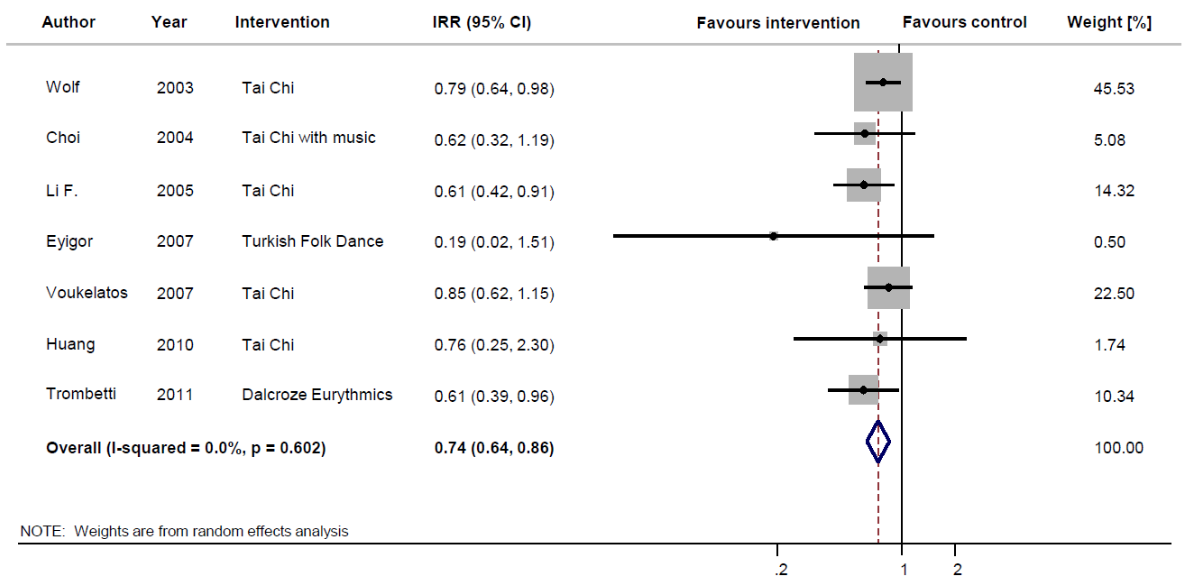

Association of dance-based mind-motor activities with rate of falls Sensitivity analysis 2: without Eyigor et al.

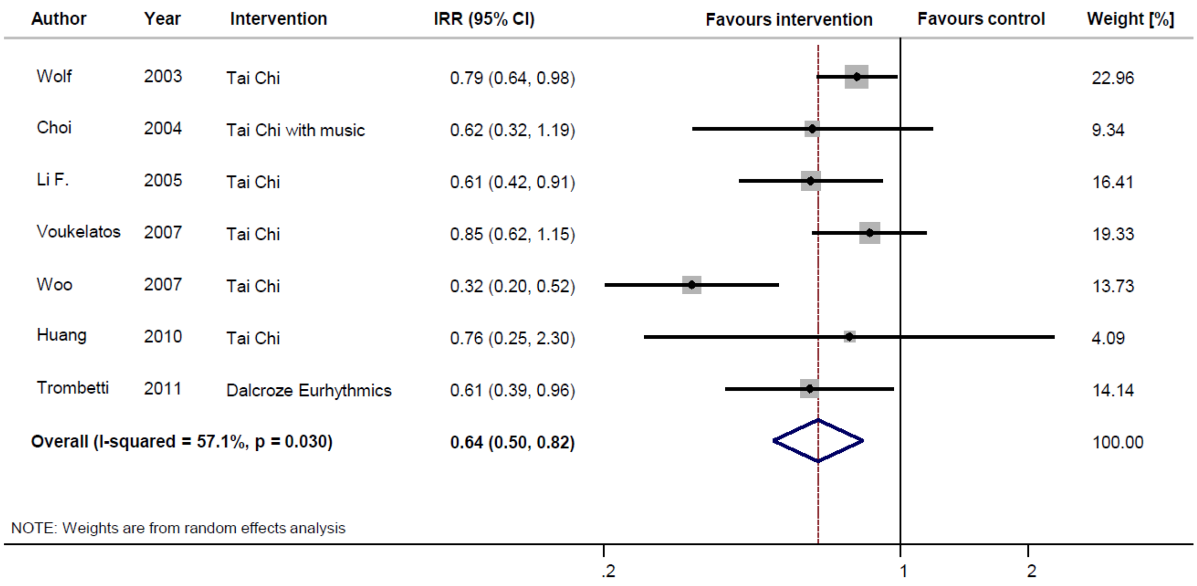

© 2020 Mattle M et al. *JAMA Network Open.*

Subgroup Analyses Rate of Falls

**Rate of Falls, total N=7 trials**	**IRR (95%CI)**	**I^2**
**Full**	**0.69 (0.53, 0.89)**	**73.0 %**
**by intervention type**		
Tai Chi	0.64 (0.54, 0.74)	0.0%
Non-Tai Chi	0.88 (0.02, 1.75)	91.7 %
**by intervention frequency**		
< 3 times / week	0.80 (0.59, 1.01)	68.0 %
≥ 3 times / week	0.55 (0.39, 0.70)	0.0%
**by intervention duration**		
> 24 weeks	1.00 (0.41, 1.60)	85.4 %
12 - 24 weeks	0.57 (0.47, 0.61)	0.0%
< 12 weeks	--	--
**by type of dwelling**		
community dwelling	0.65 (0.48, 0.82)	0.0%
institutionalized	1.00 (0.41, 1.60)	85.4 %
**by type of randomization**		
participants randomized	0.57 (0.47, 0.68)	0.0%
cluster randomized	1.00 (0.41, 1.60)	85.4 %
**by duration of one class**		
< 60 min	0.63 (0.45, 0.89)	--
≥ 60 min	0.70 (0.52, 0.95)	76.8 %
**by total contact time**		
< 90 min	0.69 (0.53, 0.89)	73.0 %
≥ 90 min	0.73 (0.53, 1.00)	79.3 %

© 2020 Mattle M et al. *JAMA Network Open.*

Assciation of dance-based mind-motor activities with rate of falls by intervention type

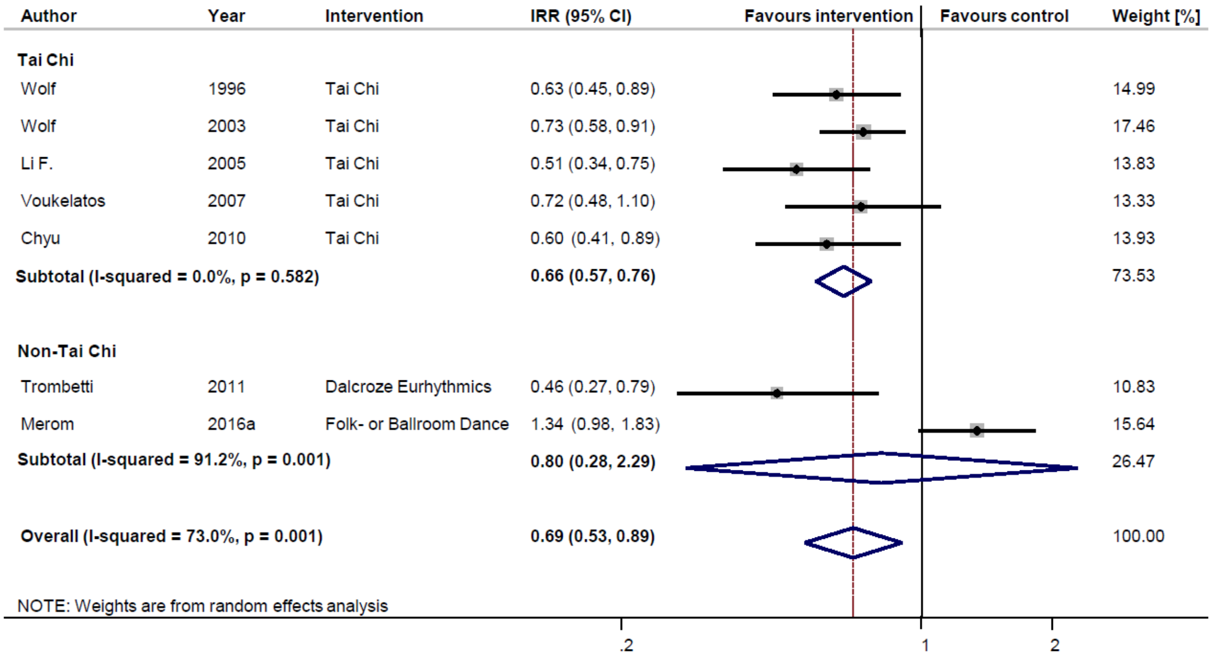

Assciation of dance-based mind-motor activities with rate of falls by intervention frequency

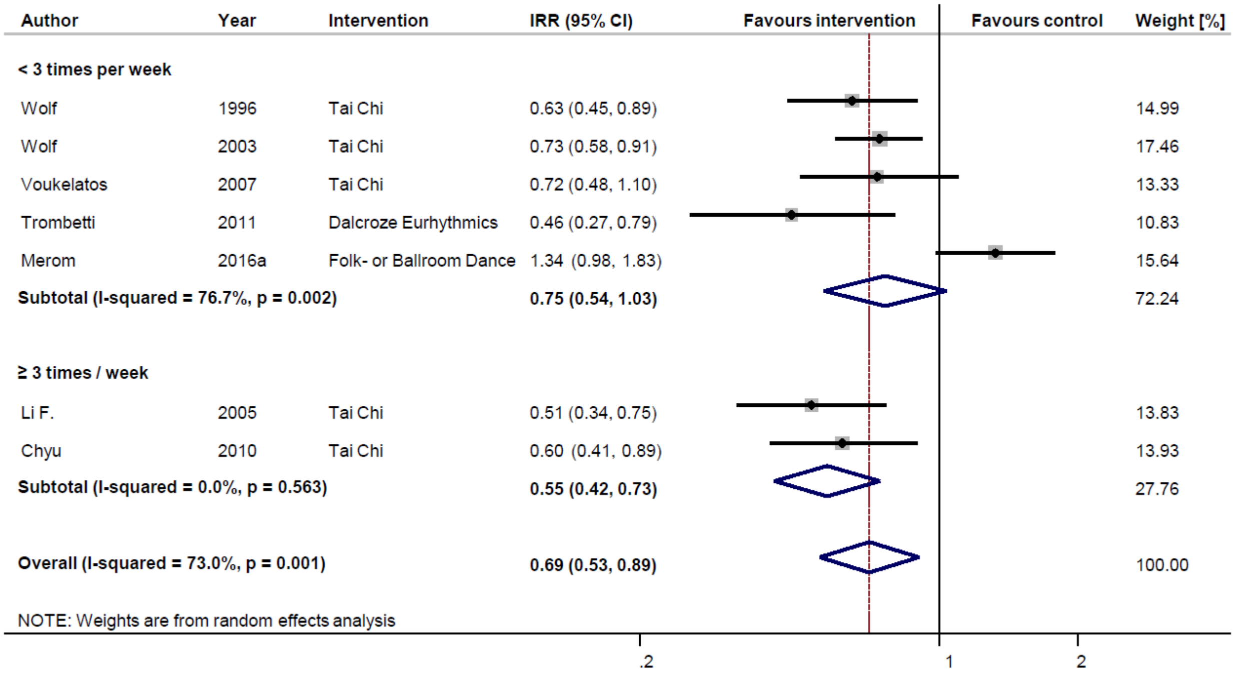

© 2020 Mattle M et al. *JAMA Network Open.*

Assciation of dance-based mind-motor activities with rate of falls by intervention duration

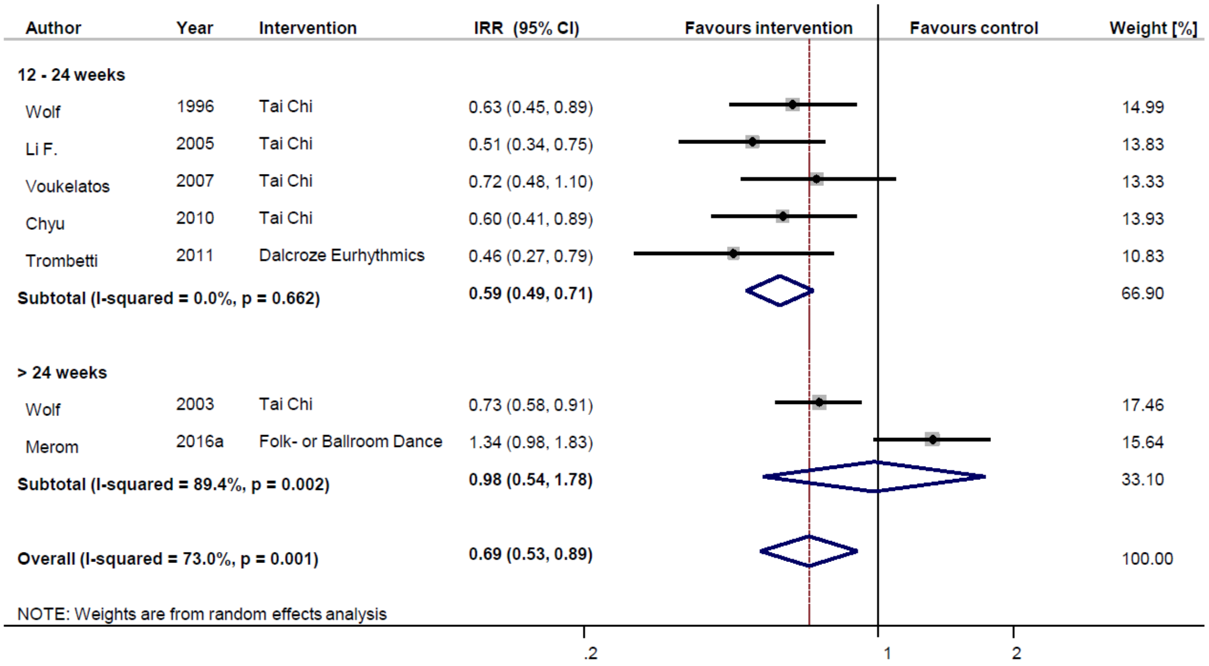

Assciation of dance-based mind-motor activities with rate of falls by type of dwelling

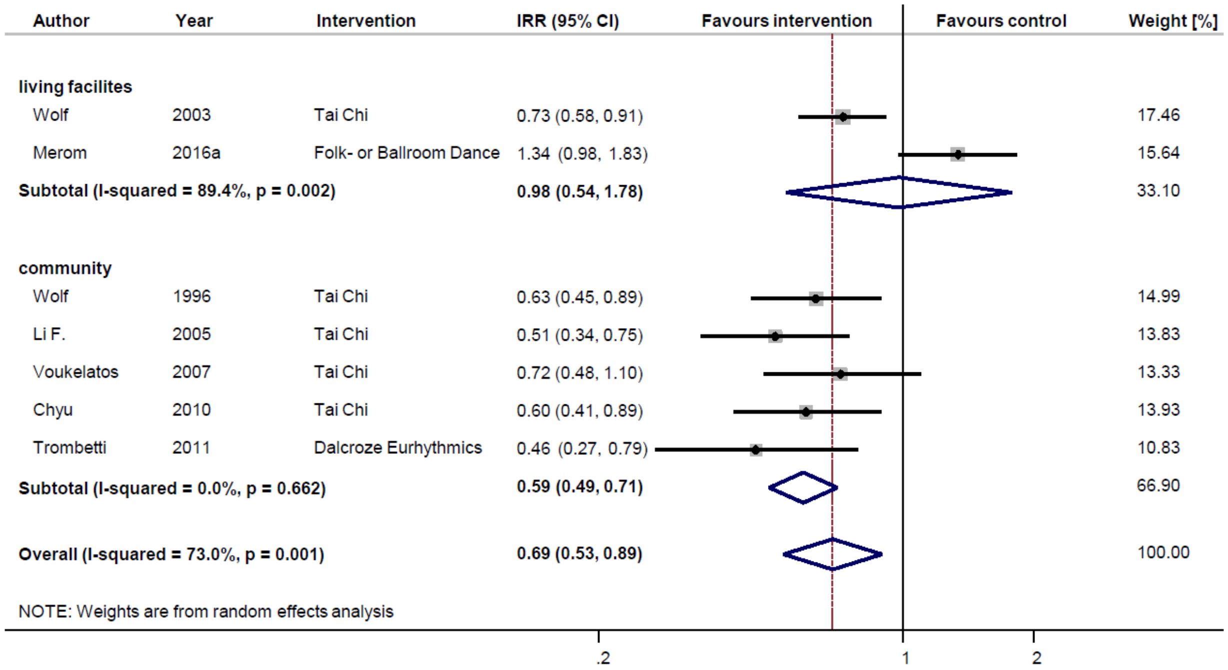

© 2020 Mattle M et al. *JAMA Network Open.*

Assciation of dance-based mind-motor activities with rate of falls by type of randomization

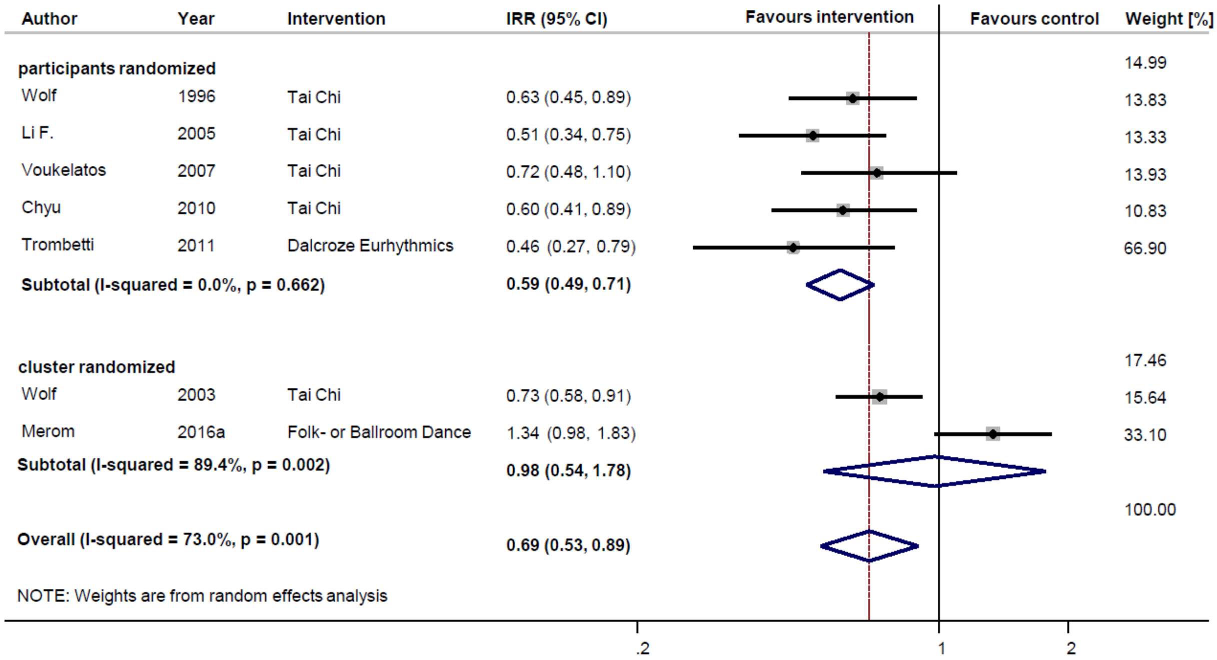

Assciation of dance-based mind-motor activities with rate of falls by duration of one class

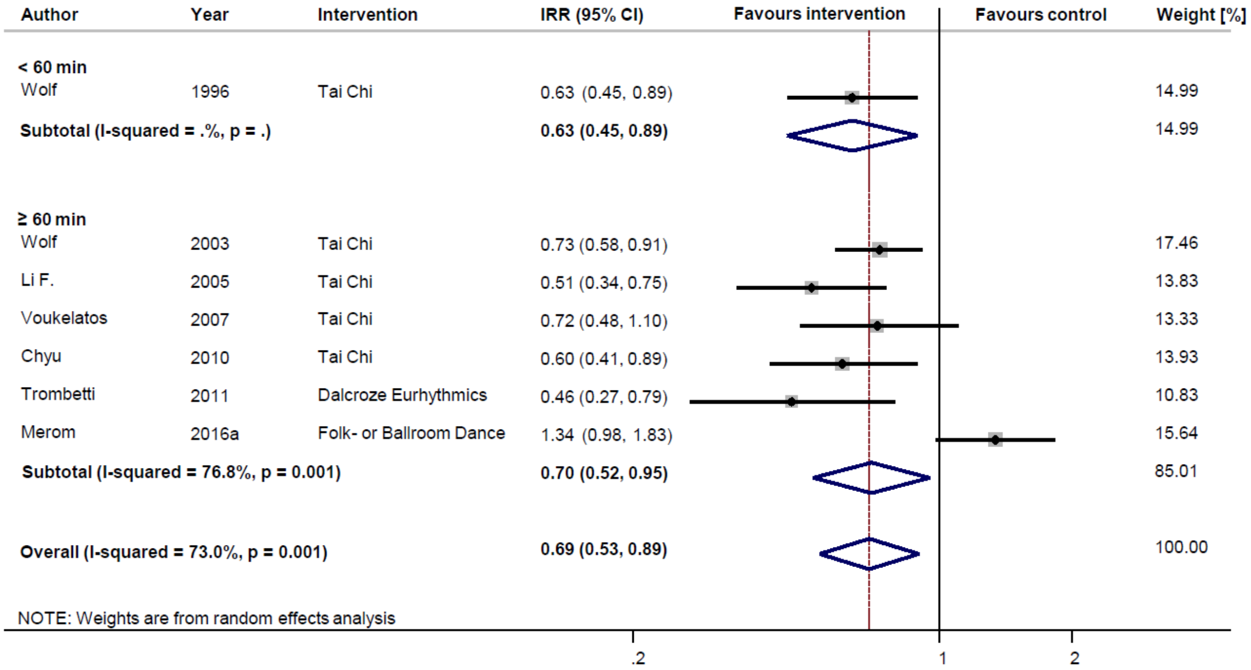

© 2020 Mattle M et al. *JAMA Network Open.*

Assciation of dance-based mind-motor activities with rate of falls by total contact time

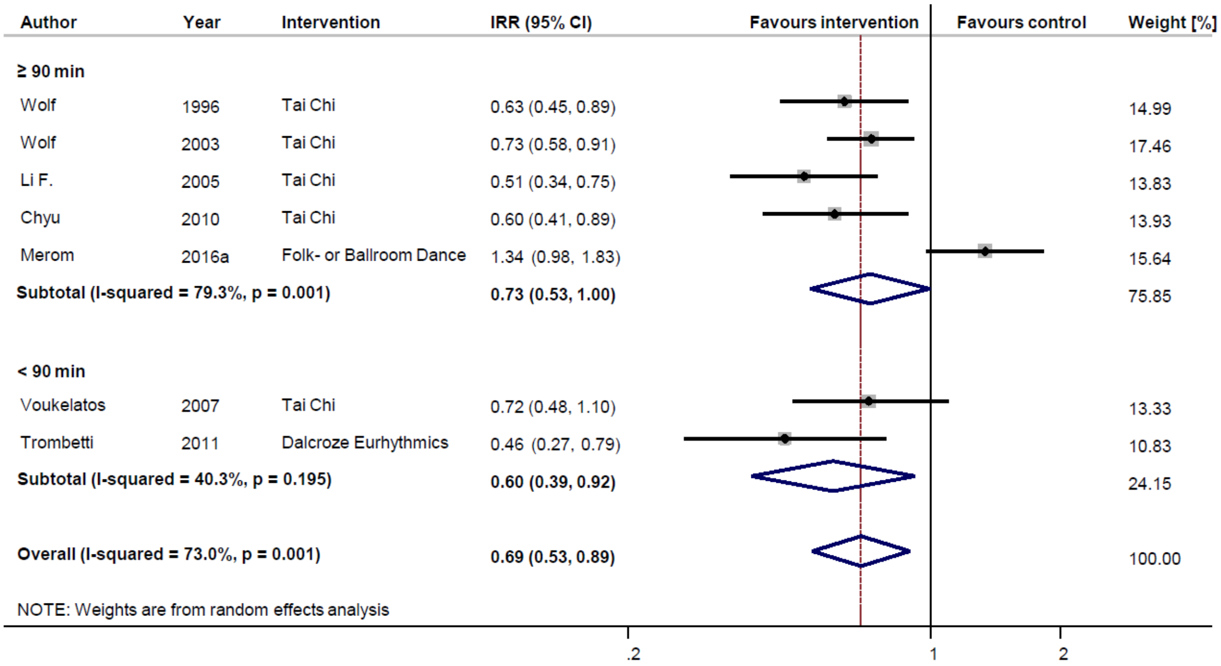

© 2020 Mattle M et al. *JAMA Network Open.*

Subgroup Analysis of Secondary Outcomes

Balance (N=15)	SMD (95%CI)	I^2
**Full**	**0.62 (0.33, 0.90)**	**90.1%**
**by intervention type**		
Tai Chi	0.62 (0.33, 0.90)	93.0 %
Non-Tai Chi	0.86 (0.33, 1.39)	81.0 %
**by intervention frequency**		
< 3 times / week	0.23 (0.02, 0.48)	62.3 %
≥ 3 times / week	0.84 (0.54, 1.14)	81.4 %
**by intervention duration**		
> 24 weeks	0.23 (0.34, 0.80)	71.7 %
12 - 24 weeks	0.54 (0.21, 0.87)	91.5 %
< 12 weeks	1.31 (0.12, 2.49)	87.4 %
**by type of dwelling**		
community dwelling	0.62 (0.314, 0.93)	90.8 %
institutionalized	0.61 0.24, 0.97)	--
**by type of randomization**		
participants randomized	0.61 (0.28, 0.93)	91.4 %
cluster randomized	0.69 (0.40, 0.98)	0.0%
**by duration of one class**		
< 60 min	0.89 (0.41, 1.36)	85.6 %
≥ 60 min	0.51 (0.10, 0.92)	92.7 %
Woo et al. do not report time		
**by total contact time**		
< 90 min	0.49 (0.15, 0.83)	--
90 -120 min	0.33 (0.02, 0.64)	76.6 %
≥ 120 min	0.96 (0.64, 1.28)	76.7 %
Woo et al. do not report duration of classes		

© 2020 Mattle M et al. *JAMA Network Open.*

Association of dance-based mind-motor activities with balance by intervention type

Author	Year	Intervention	SMD (95% CI)	Weight [%]
**Tai Chi**				
Choi	2004	Tai Chi with music	0.61 (0.24, 0.97)	7.14
Li F.	2005	Tai Chi	1.01 (0.91, 1.12)	8.00
Woo	2007	Tai Chi	-0.09 (-0.59, 0.41)	6.48
Li J.	2008	Tai Chi	0.91 (0.54, 1.28)	7.11
Pereira	2008	Tai Chi	1.02 (0.55, 1.49)	6.63
Logghe	2009	Tai Chi	-0.04 (-0.27, 0.20)	7.66
Huang	2010	Tai Chi	0.83 (0.36, 1.29)	6.65
Taylor-Piliae	2010	Tai Chi	0.26 (-0.03, 0.56)	7.44
Sun	2015	Tai Chi	-0.12 (-0.45, 0.21)	7.29
**Subtotal (I-squared = 93.0%, p = 0.000)**			**0.49 (0.12, 0.85)**	64.40
**Non-Tai Chi**				
Hopkins	1990	low-impact Aerobic Dance	0.48 (-0.06, 1.02)	6.26
Eyigor	2007	Turkish Dance	0.61 (-0.04, 1.25)	5.72
Trombetti	2011	Dalcroze Eurythmics	0.49 (0.15, 0.83)	7.25
Alves	2013	Ballroom Dance	0.49 (-0.07, 1.04)	6.20
Serrano-Guzman	2016	Flamenco and Sevillanas	2.47 (1.76, 3.19)	5.36
Bennett	2018	Line Dancing	0.83 (0.01, 1.66)	4.83
**Subtotal (I-squared = 81.0%, p = 0.000)**			**0.86 (0.33, 1.39)**	35.60
**Overall (I-squared = 90.1%, p = 0.000)**			**0.62 (0.33, 0.90)**	100.00

NOTE: Weights are from random effects analysis

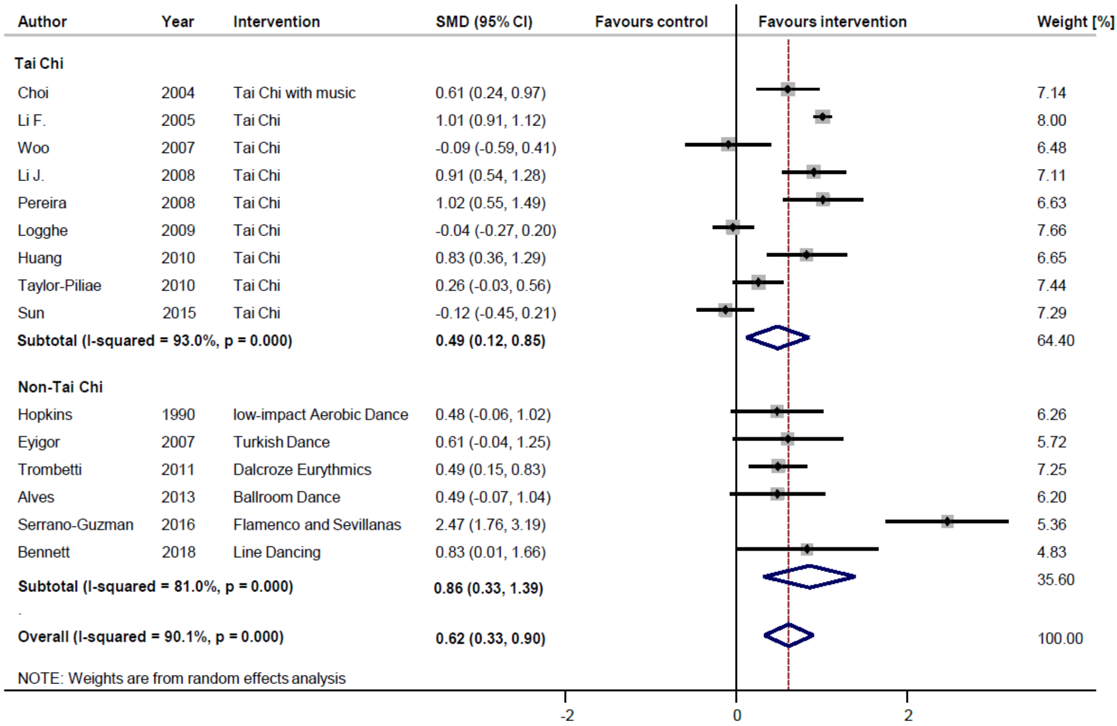

Association of dance-based mind-motor activities with balance by intervention frequency

Author	Year	Intervention	SMF (95% CI)	Weight [%]
**< 3 times / week**				
Logghe	2009	Tai Chi	-0.04 (-0.27, 0.20)	7.66
Taylor-Piliae	2010	Tai Chi	0.26 (-0.03, 0.56)	7.44
Trombetti Alves	2011	Dalcroze Eurythmics	0.49 (0.15, 0.83)	7.25
Sun	2013	Ballroom Dance	0.49 (-0.07, 1.04)	6.20
Bennett	2015	Tai Chi	-0.12 (-0.45, 0.21)	7.29
	2018	Line Dancing	0.83 (0.01, 1.66)	4.83
**Subtotal (I-squared = 62.3%, p = 0.021)**			**0.23 (-0.02, 0.48)**	40.66
**≥ 3 times / week**				
Hopkins	1990	low-impact Aerobic Dance	0.48 (-0.06, 1.02)	6.26
Choi	2004	Tai Chi with music	0.61 (0.24, 0.97)	7.14
Li F.	2005	Tai Chi	1.01 (0.91, 1.12)	8.00
Eyigor	2007	Turkish Dance	0.61 (-0.04, 1.25)	5.72
Woo	2007	Tai Chi	-0.09 (-0.59, 0.41)	6.48
Li J.	2008	Tai Chi	0.91 (0.54, 1.28)	7.11
Pereira	2008	Tai Chi	1.02 (0.55, 1.49)	6.63
Huang	2010	Tai Chi	0.83 (0.36, 1.29)	6.65
Serrano-Guzman	2016	Flamenco and Sevillanas	2.47 (1.76, 3.19)	5.36
**Subtotal (I-squared = 81.4%, p = 0.000)**			**0.84 (0.54, 1.14)**	59.34
**Overall (I-squared = 90.1%, p = 0.000)**			**0.62 (0.33, 0.90)**	100.00

NOTE: Weights are from random effects analysis

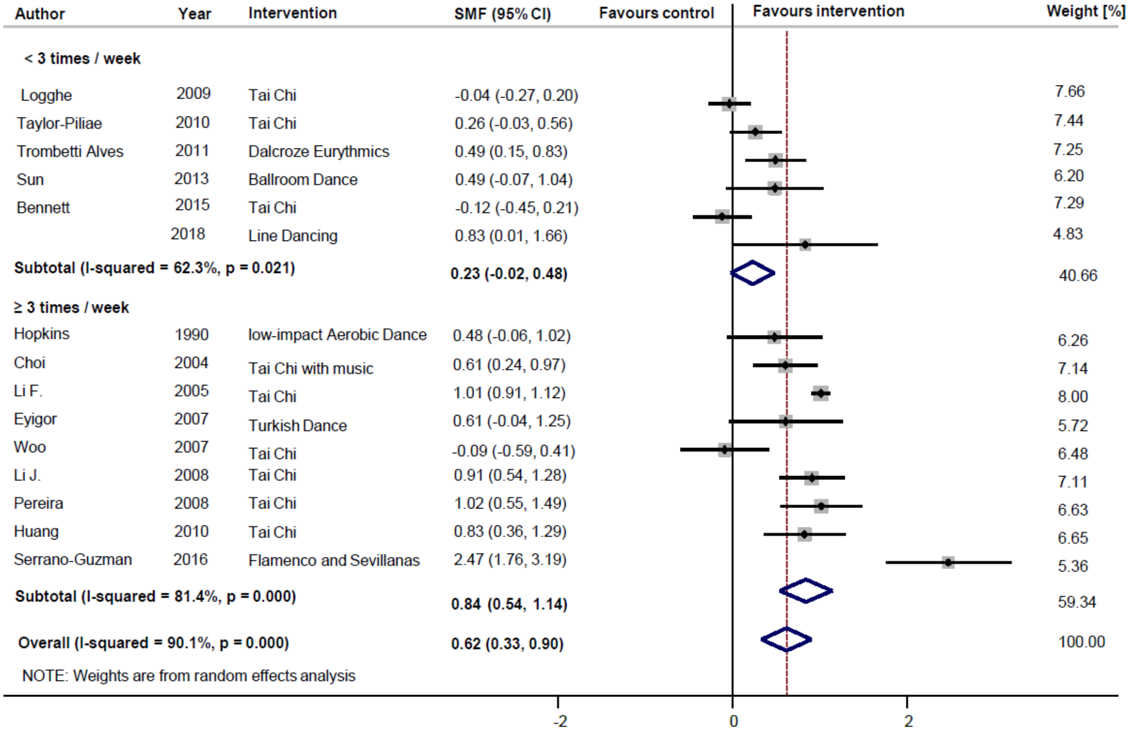

© 2020 Mattle M et al. *JAMA Network Open.*

Association of dance-based mind-motor activities with balance by intervention duration

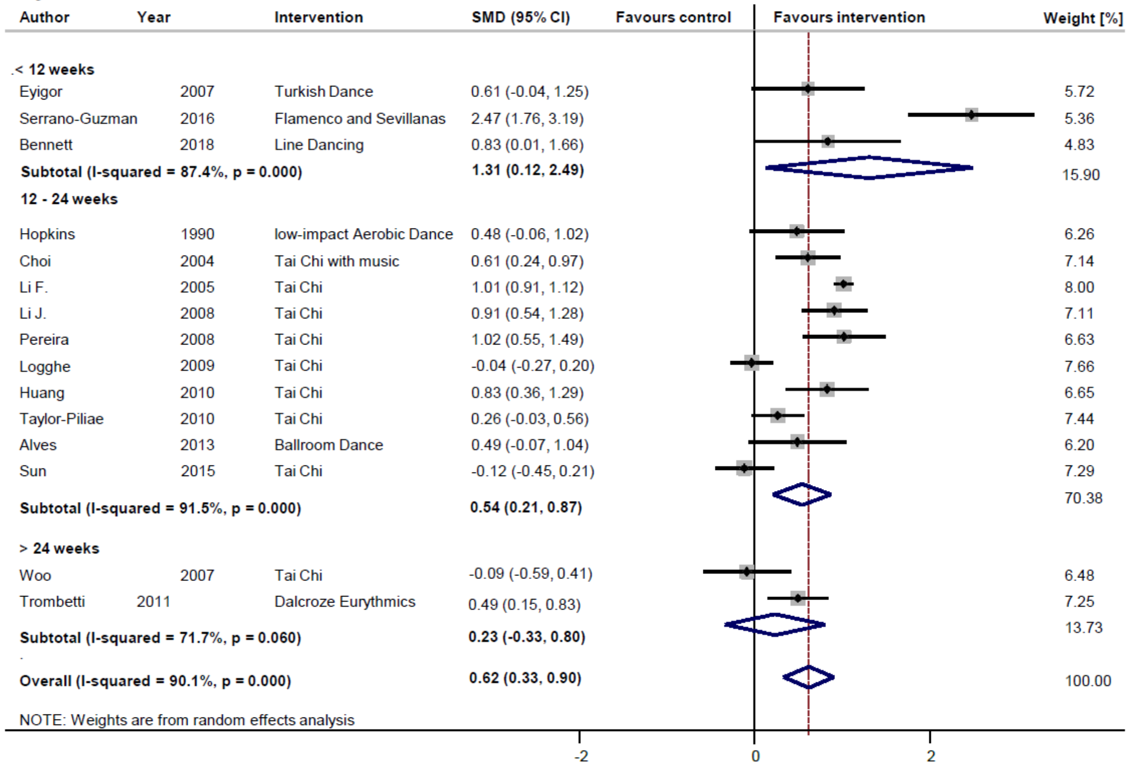

Association of dance-based mind-motor activities with balance by type of randomization

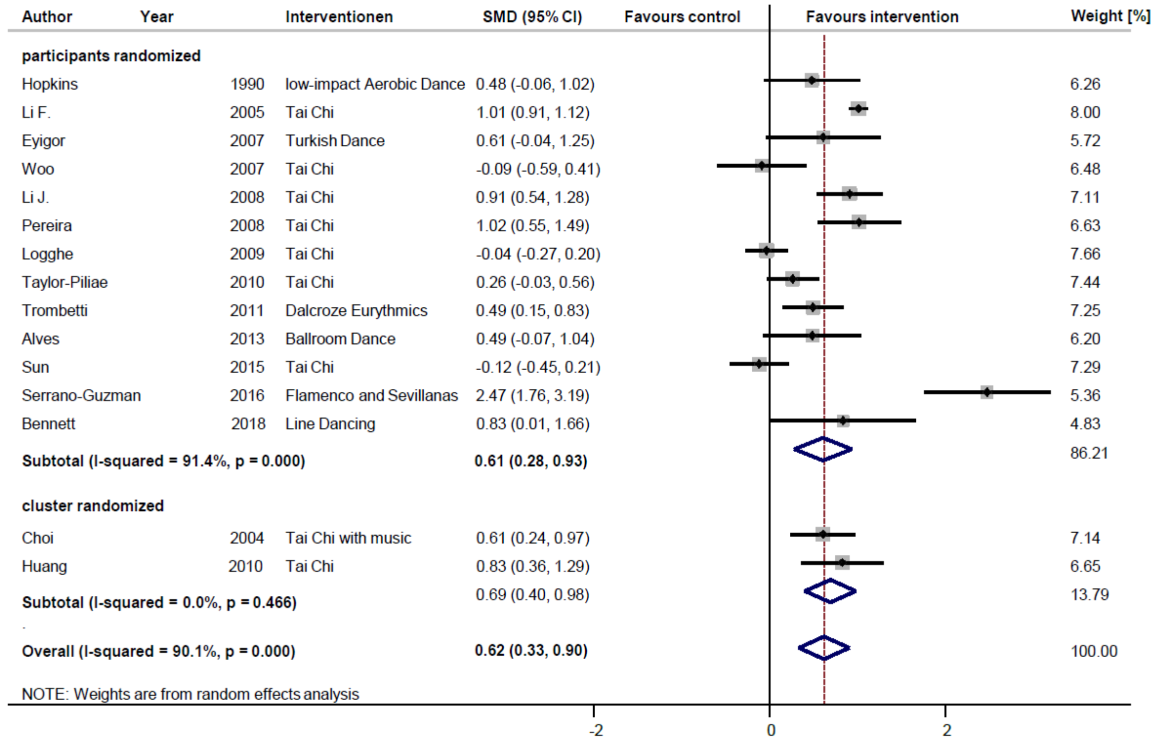

© 2020 Mattle M et al. *JAMA Network Open.*

Association of dance-based mind-motor activities with balance by duration of one class

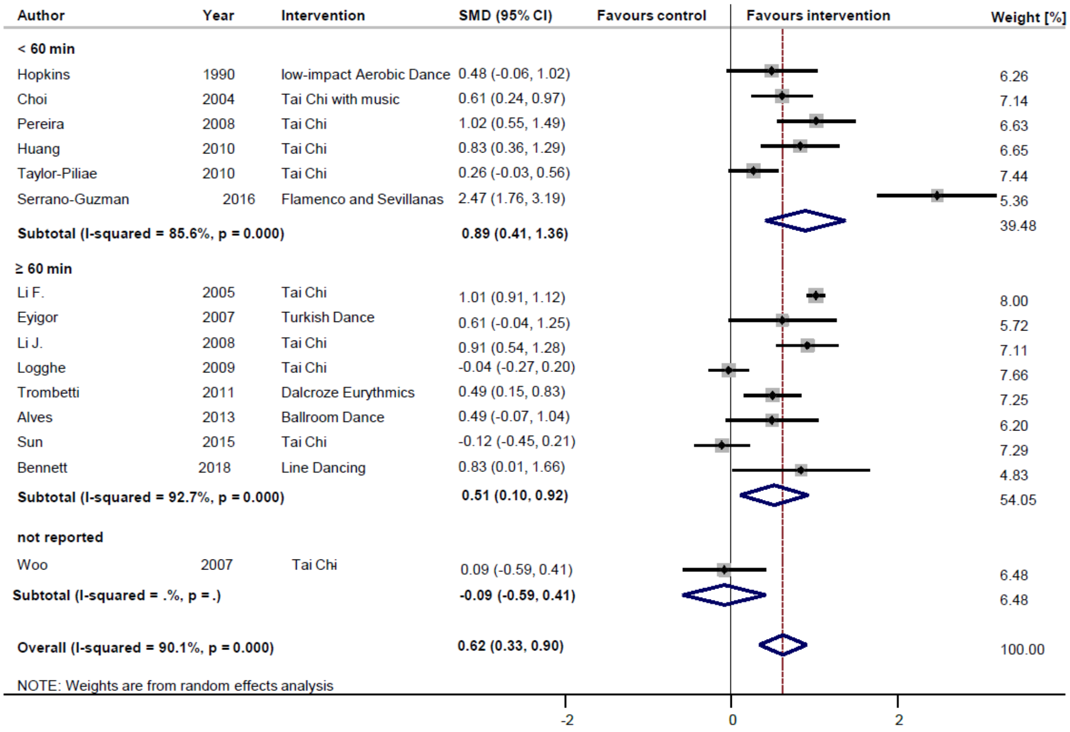

Association of dance-based mind-motor activities with balance by total contact time

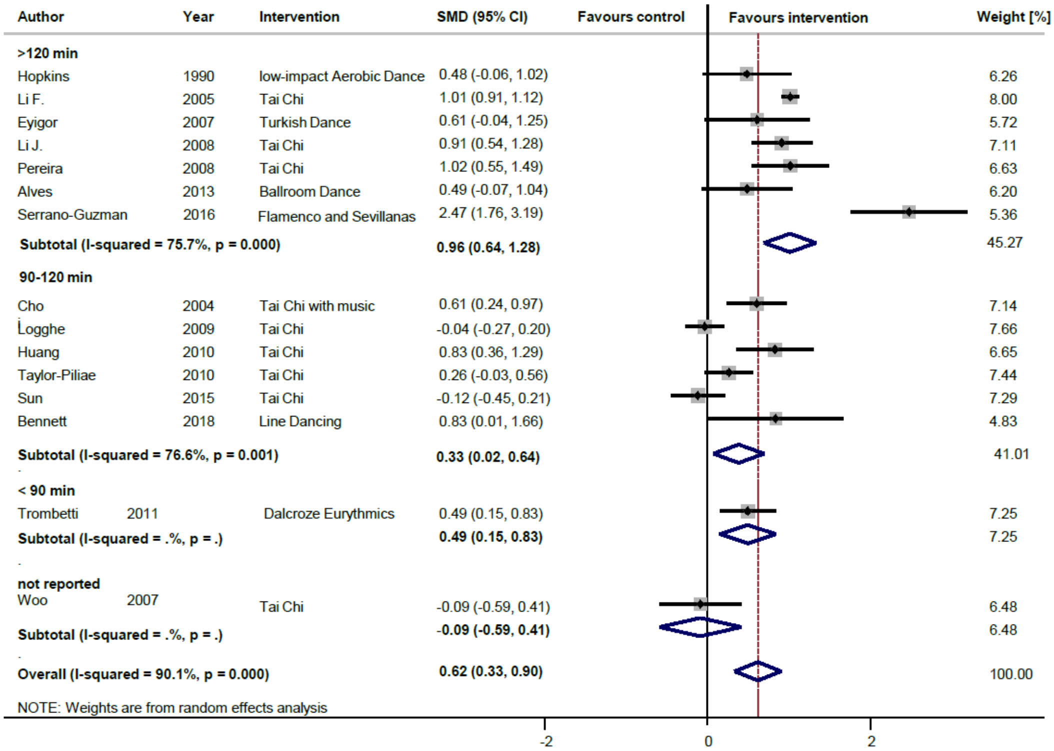

© 2020 Mattle M et al. *JAMA Network Open.*

Mobility (N=13)	SMD (95%CI)	I^2
**Full**	**-0.56 (-0.81, -0.31)**	**77.1%**
**by intervention type**		
Tai Chi	-0.31 (0.60, -0.01)	70.3%
Non-Tai Chi	-0.79, (-1.16, -0.42)	72.5%
**by intervention frequency**		
< 3 times / week	-0.231 (-0.46, -0.01)	41.4%
≥ 3 times / week	-0.762 (-1.10, 0.42)	73.1%
**by intervention duration**		
> 24 weeks	-0.26 (-0.60, 0.08)	--
12 - 24 weeks	-0.44 (-0.68, -0.20)	69.7%
< 12 weeks	-1.46 (-1.96, -0.96)	10.4%
**by type of dwelling**		
community dwelling	-0.56 (-0.81, -0.31)	77.1%
institutionalized	--	--
**by type of randomization**		
participants randomized	-0.59 (-0.89, -0.30)	80.4%
cluster randomized	-0.44 (-0.76, -0.11)	12.6%
**by duration of one class**		
< 60 min	-0.75 (-1.09, -0.42)	55.0%
≥ 60 min	-0.43 (-0.73, -0.12)	77.9%
**by total contact time**		
< 90 min	-0.26 (-0.60, 0.07)	--
90-120 min	-0.25 (-0.61, 0.10)	68.8%
> 120 min	-0.73 (-1.05, -0.40)	69.3%

© 2020 Mattle M et al. *JAMA Network Open.*

Association of dance-based mind-motor activities with mobility by intervention type

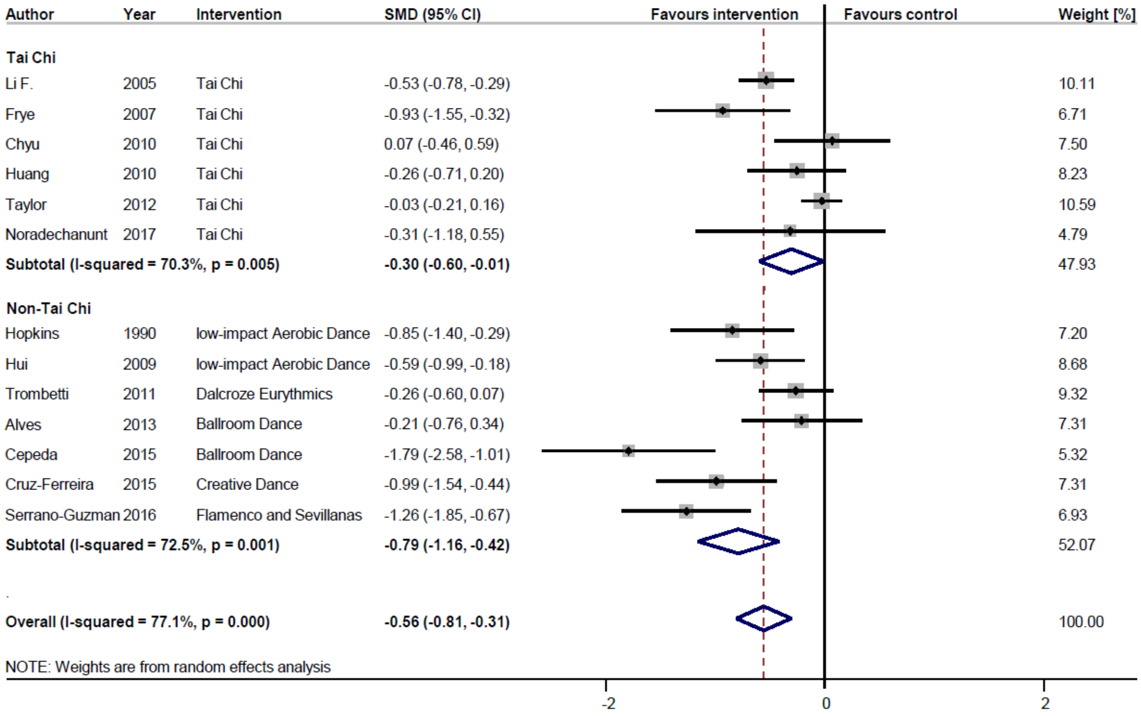

Association of dance-based mind-motor activities with mobility by intervention frequency

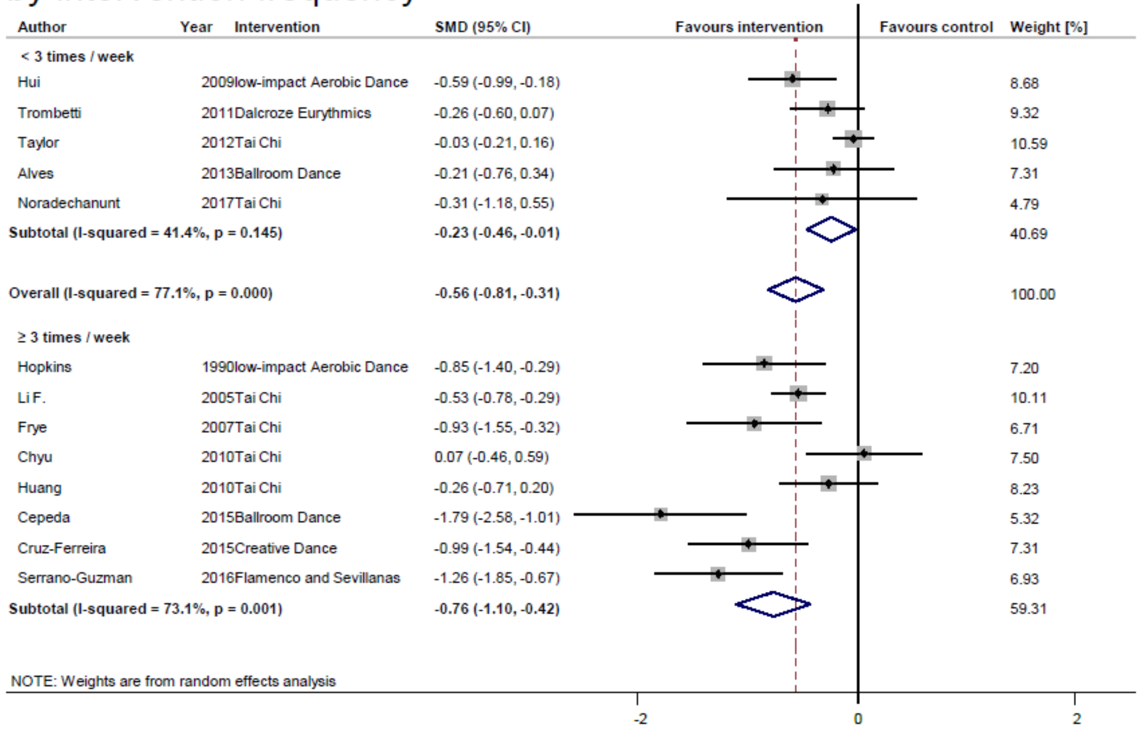

© 2020 Mattle M et al. *JAMA Network Open.*

Association of dance-based mind-motor activities with mobility by intervention duration

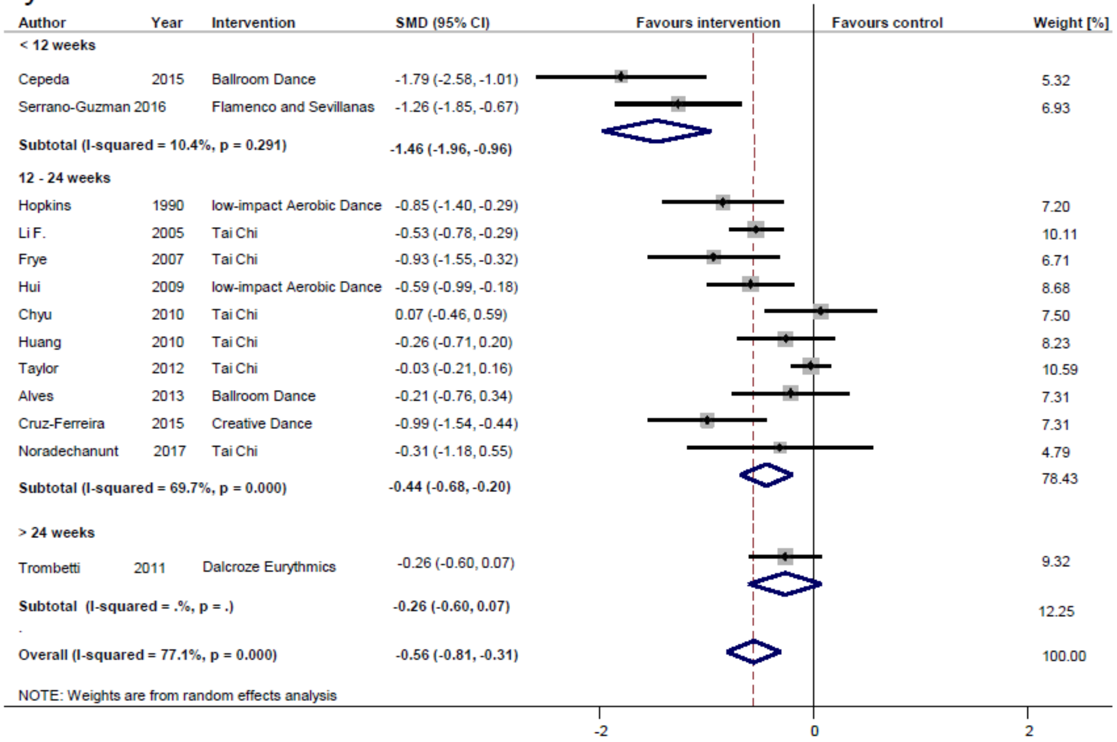

Association of dance-based mind-motor activities with mobility by type of randomization

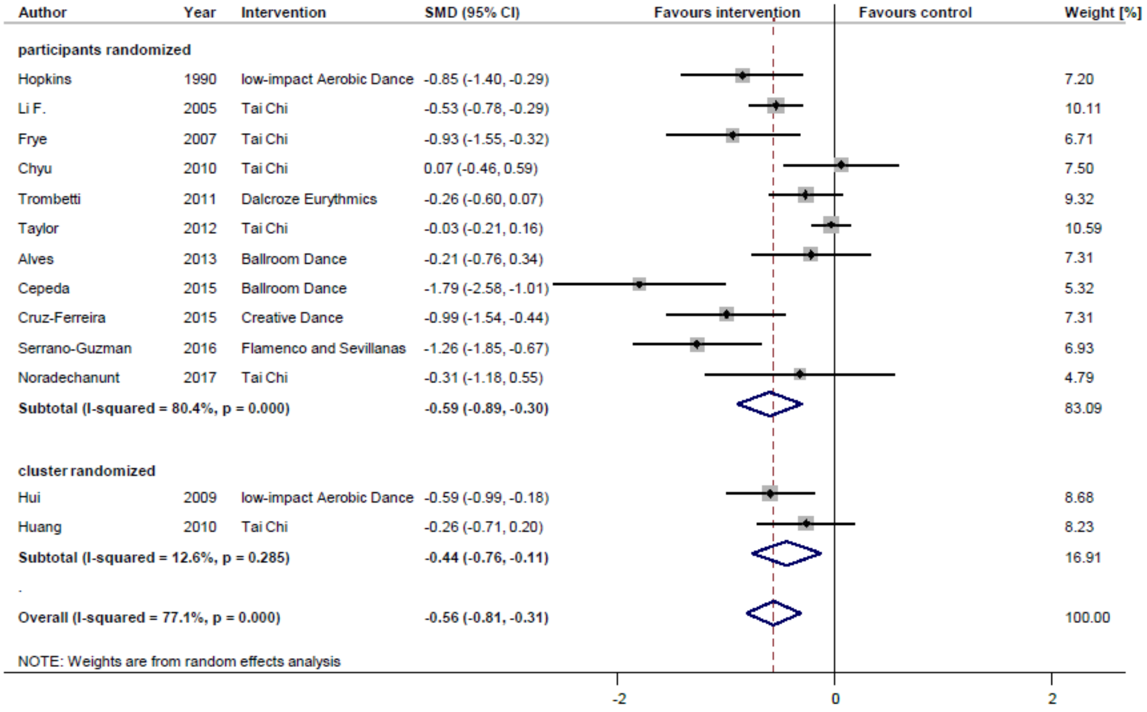

© 2020 Mattle M et al. *JAMA Network Open.*

Association of dance-based mind-motor activities with mobility by duration of one class

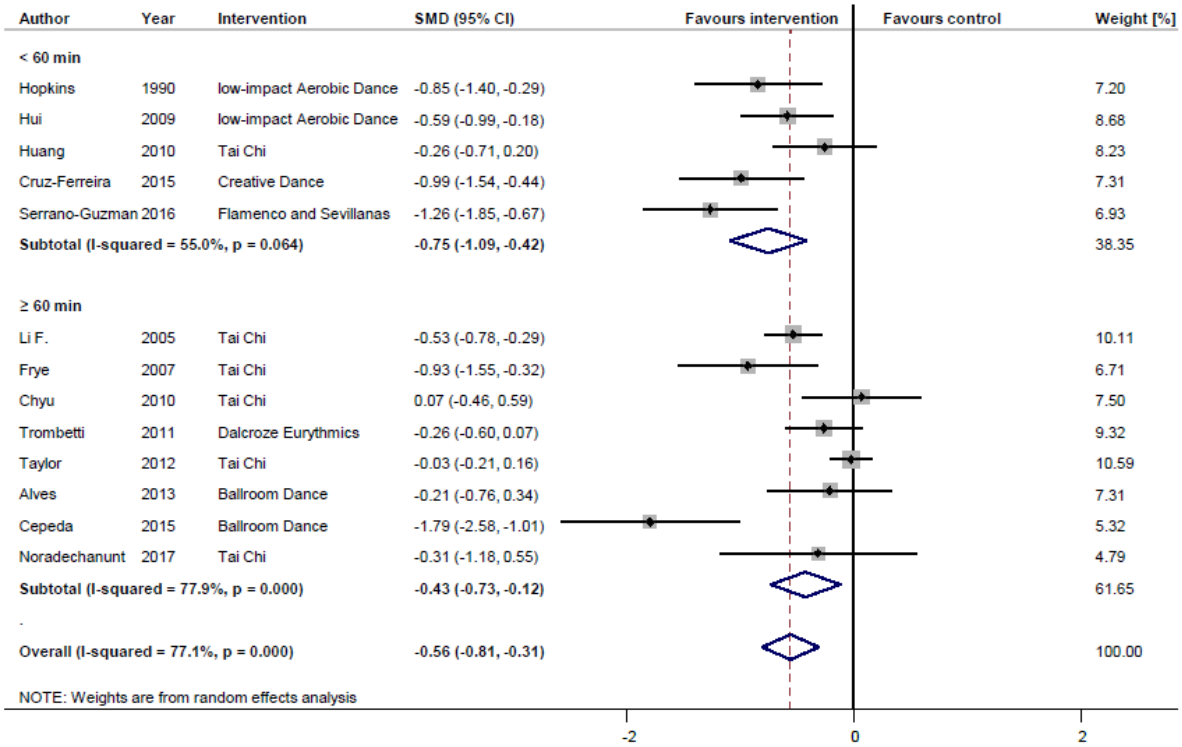

Association of dance-based mind-motor activities with mobility by total contact time

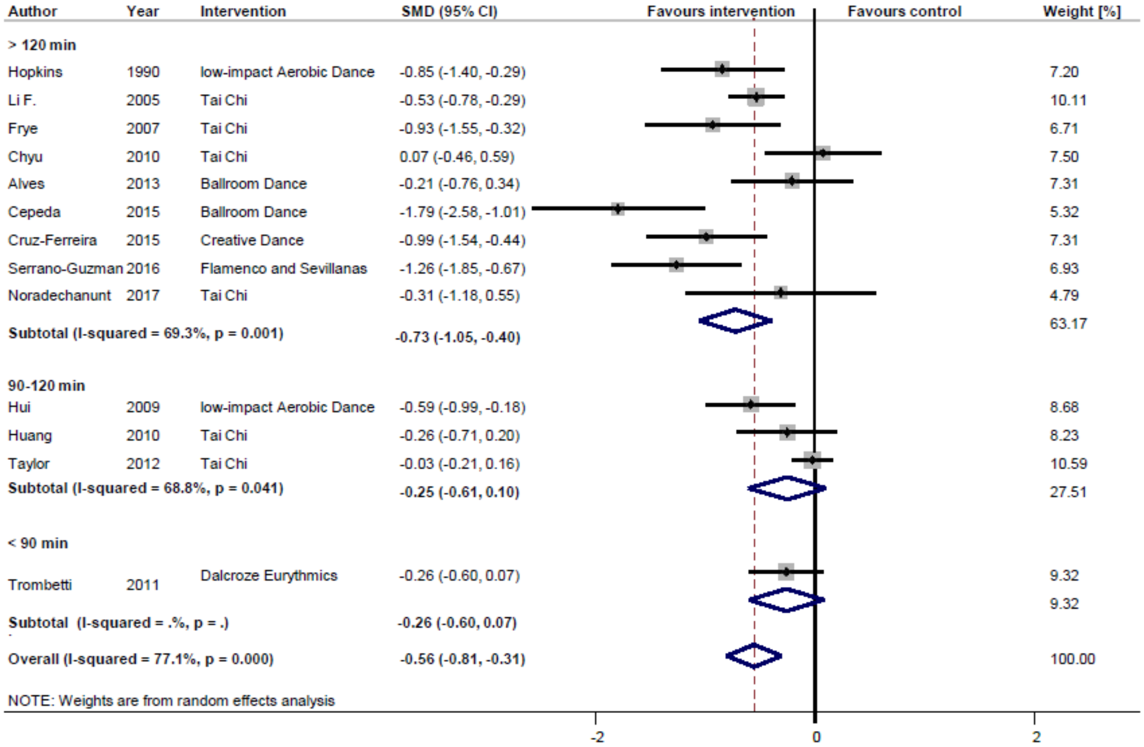

© 2020 Mattle M et al. *JAMA Network Open.*

**Strength (lower body) (N=13)**	**SMD (95%CI)**	**I^2**
**Full**	**0.57 (0.23, 0.91)**	**88.5%**
**by intervention type**		
Tai Chi	0.13 (-0.09, 0.34)	25.3%
Non-Tai Chi	0.86 (0.25, 1.47)	92.8%
**by intervention frequency**		
< 3 times / week	0.19 (-0.09, 0.46)	75.6%
≥ 3 times / week	1.04 (0.31, 1.77)	88.1%
**by intervention duration**		
> 24 weeks	-0.17 (0.33, -0.01)	0.0%
12 - 24 weeks	0.61 (0.13, 1.09)	88.8%
< 12 weeks	1.15 (0.73, 1.57)	0.0%
**by type of dwelling**		
community dwelling	0.66 (0.27, 1.05)	86.9%
institutionalized	-0.18 (-0.35, -0.01)	--
**by type of randomization**		
participants randomized	0.66 (0.23, 1.08)	87.4%
cluster randomized	0.25 (0.63, 1.13	93.6%
**by duration of one class**		
< 60 min	1.13 (0.46, 1.81)	87.7%
≥ 60 min	0.16 (-0.10, 0.41)	68.7%
**by total contact time**		
90 - 120 min	0.27 (-0.06, 0.61)	85.7%
> 120 min	0.86 (0.21, 1.51)	85.3%

© 2020 Mattle M et al. *JAMA Network Open.*

Association of dance-based mind-motor activities with LBS by intervention type

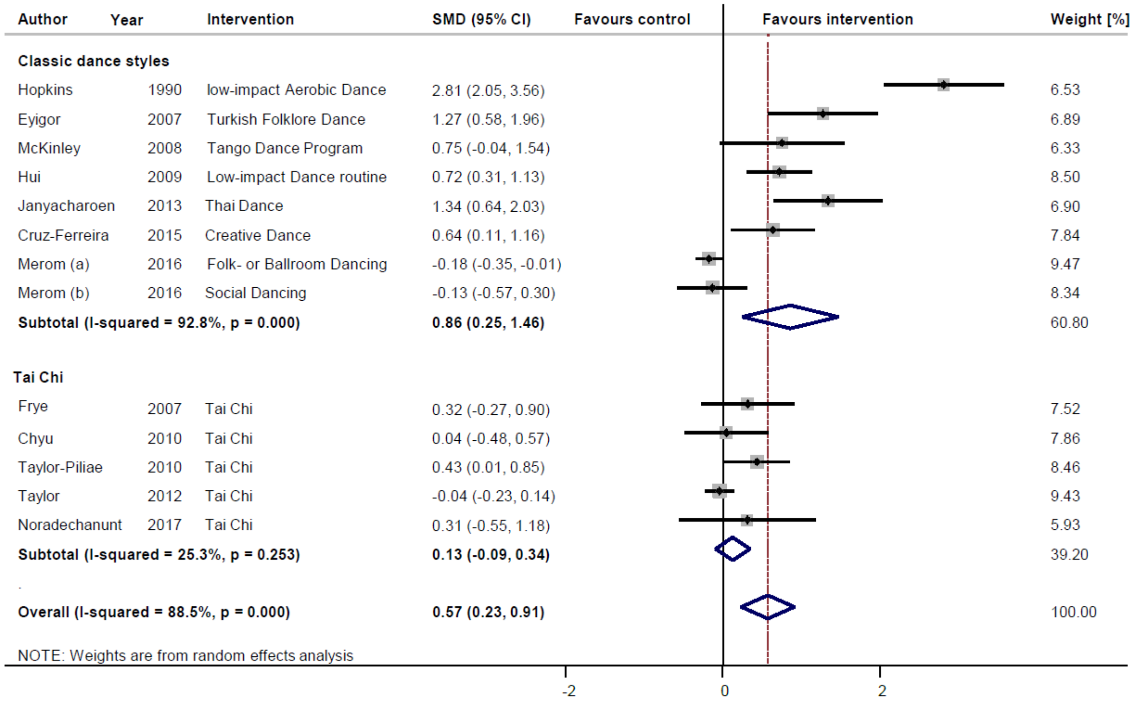

Association of dance-based mind-motor activities with LBS by intervention frequency

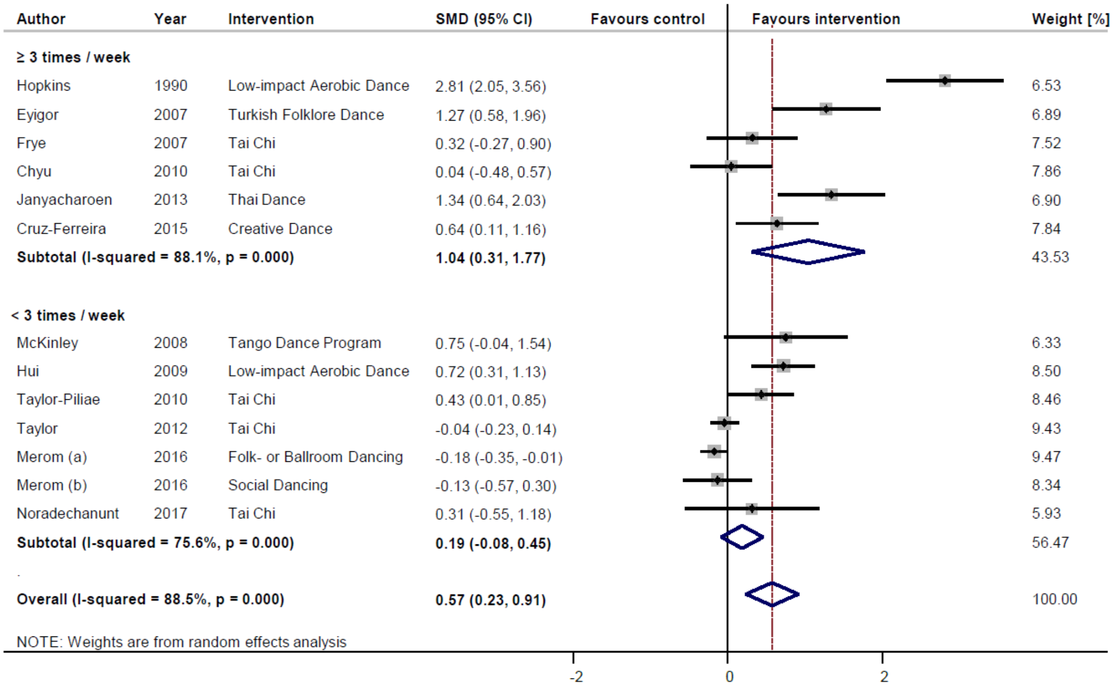

© 2020 Mattle M et al. *JAMA Network Open.*

Association of dance-based mind-motor activities with LBS by intervention duration

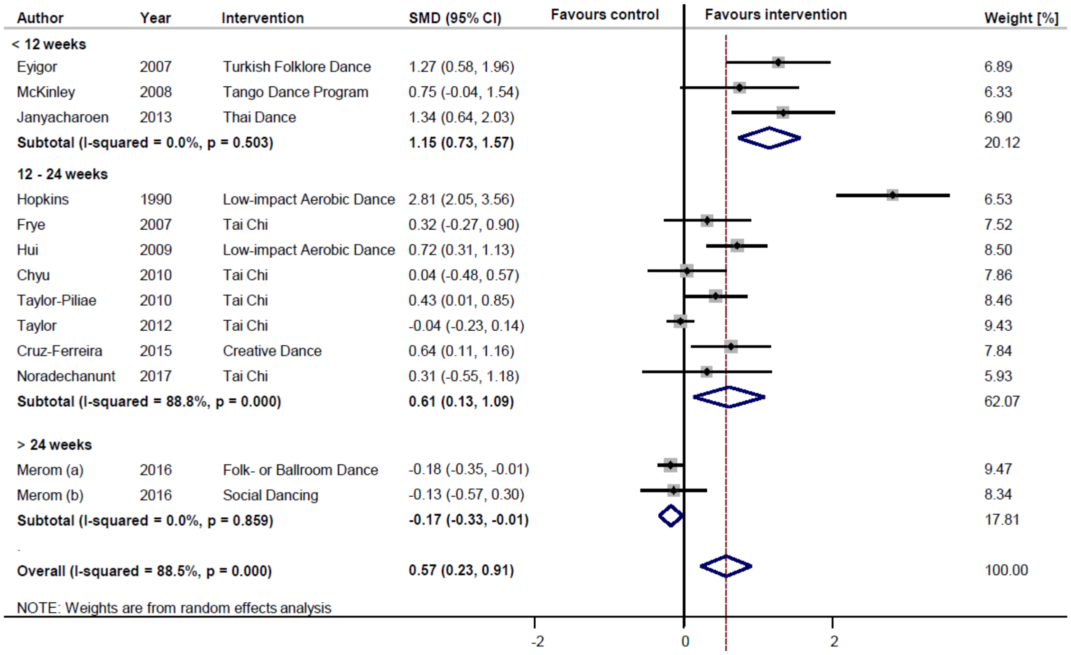

Association of dance-based mind-motor activities with LBS by type of dwelling

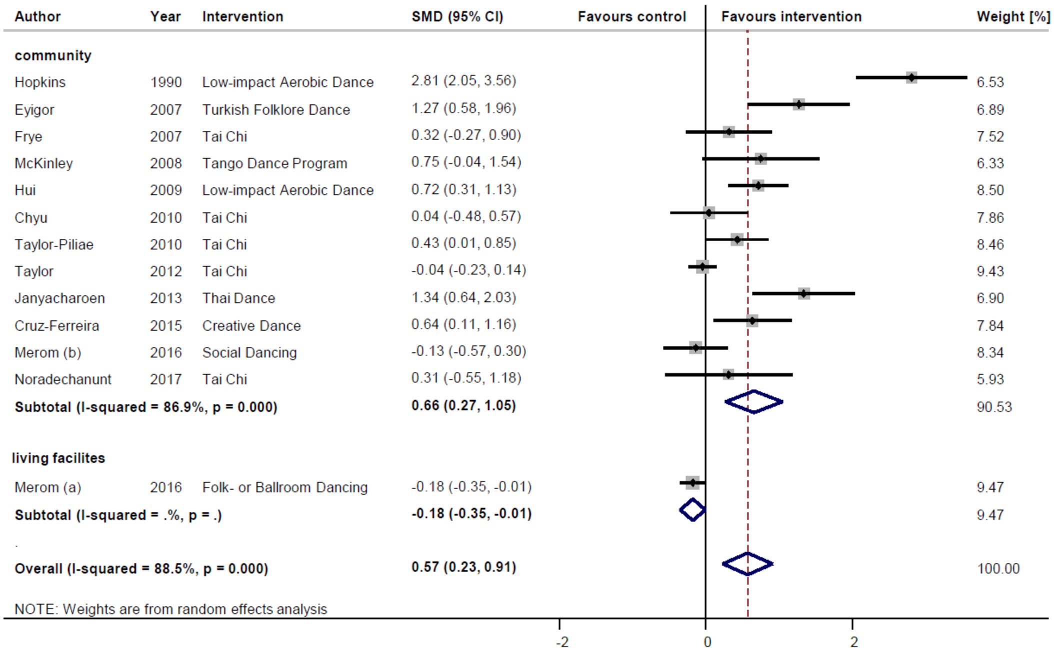

© 2020 Mattle M et al. *JAMA Network Open.*

Association of dance-based mind-motor activities with LBS by type of randomization

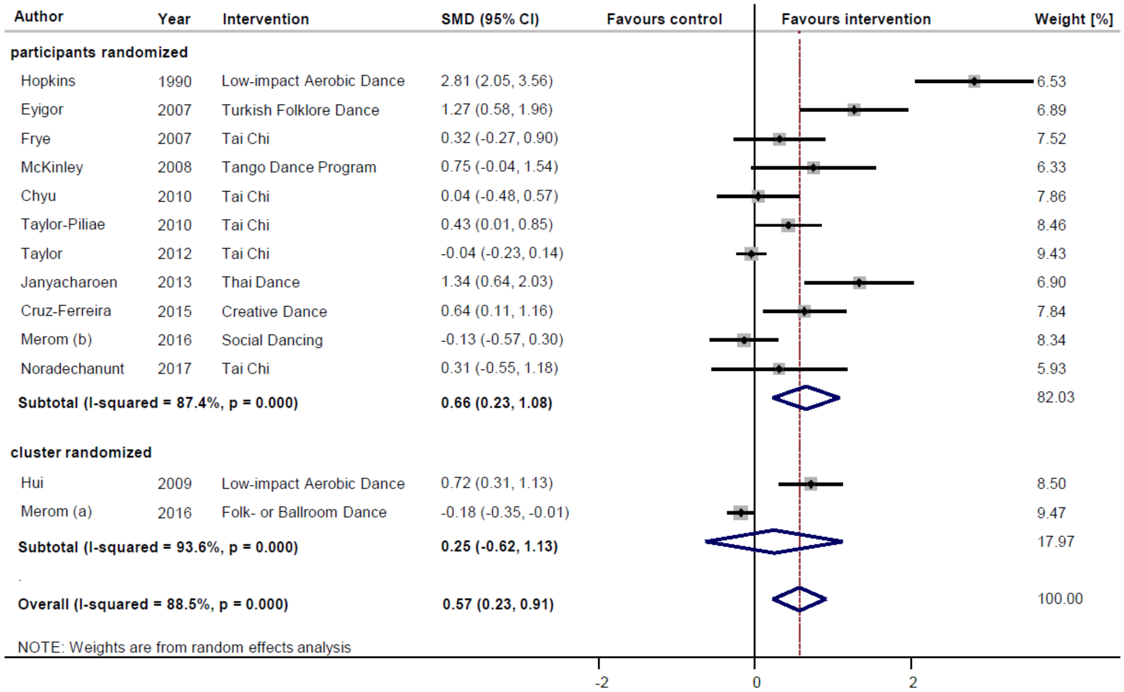

Association of dance-based mind-motor activities with LBS by duration of one class

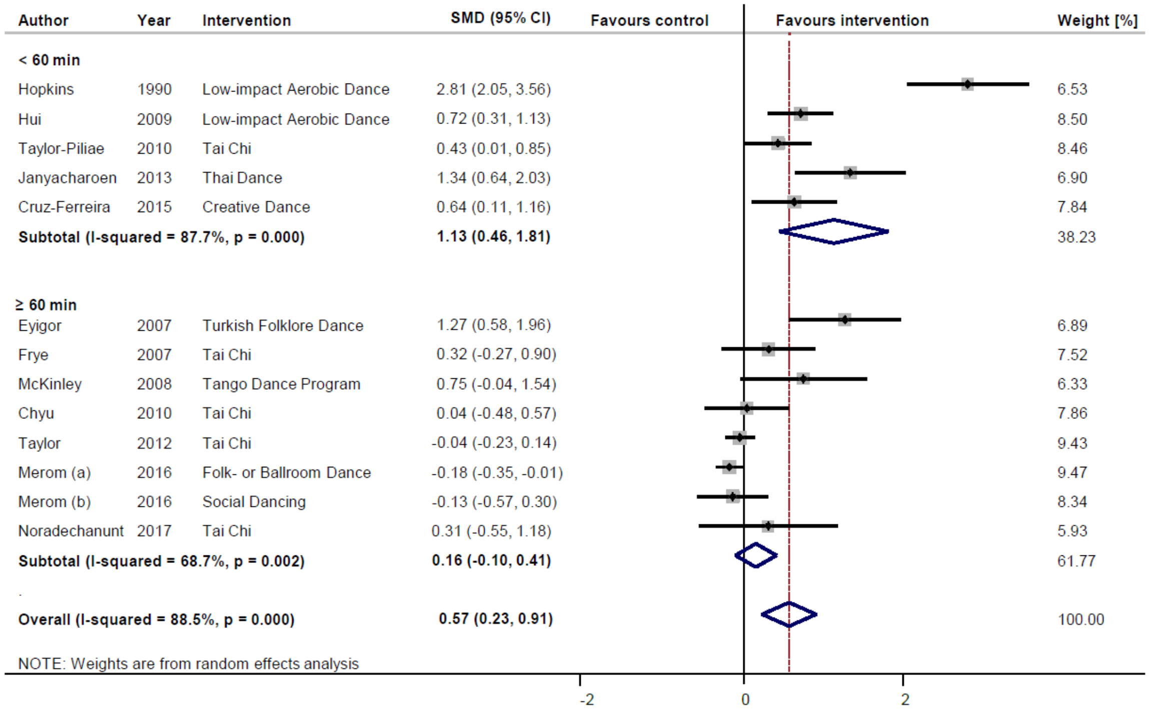

© 2020 Mattle M et al. *JAMA Network Open.*

Association of dance-based mind-motor activities with LBS by total contact time

Author	Year	Intervention	SMD (95% CI)	Weight [%]
**> 120 min**				
Hopkins	1990	Low-impact Aerobic Dance	2.81 (2.05, 3.56)	6.53
Eyigor	2007	Turkish Folklore Dance	1.27 (0.58, 1.96)	6.89
Frye	2007	Tai Chi	0.32 (-0.27, 0.90)	7.52
McKinley	2008	Tango Dance Program	0.75 (-0.04, 1.54)	6.33
Chyu	2010	Tai Chi	0.04 (-0.48, 0.57)	7.86
Cruz-Ferreira	2015	Creative Dance	0.64 (0.11, 1.16)	7.84
Noradechanunt	2017	Tai Chi	0.31 (-0.55, 1.18)	5.93
**Subtotal (I-squared = 85.3%, p = 0.000)**			**0.86 (0.21, 1.51)**	48.90
**90-120 min**				
Hui	2009	Low-impact Aerobic Dance	0.72 (0.31, 1.13)	8.50
Taylor-Piliae	2010	Tai Chi	0.43 (0.01, 0.85)	8.46
Taylor	2012	Tai Chi	-0.04 (-0.23, 0.14)	9.43
Janyacharoen	2013	Thai Dance	1.34 (0.64, 2.03)	6.90
Merom (a)	2016	Folk- or Ballroom Dancing	-0.18 (-0.35, -0.01)	9.47
Merom (b)	2016	Social Dancing	-0.13 (-0.57, 0.30)	8.34
**Subtotal (I-squared = 85.7%, p = 0.000)**			**0.27 (-0.06, 0.61)**	51.10
**Overall (I-squared = 88.5%, p = 0.000)**			**0.57 (0.23, 0.91)**	100.00

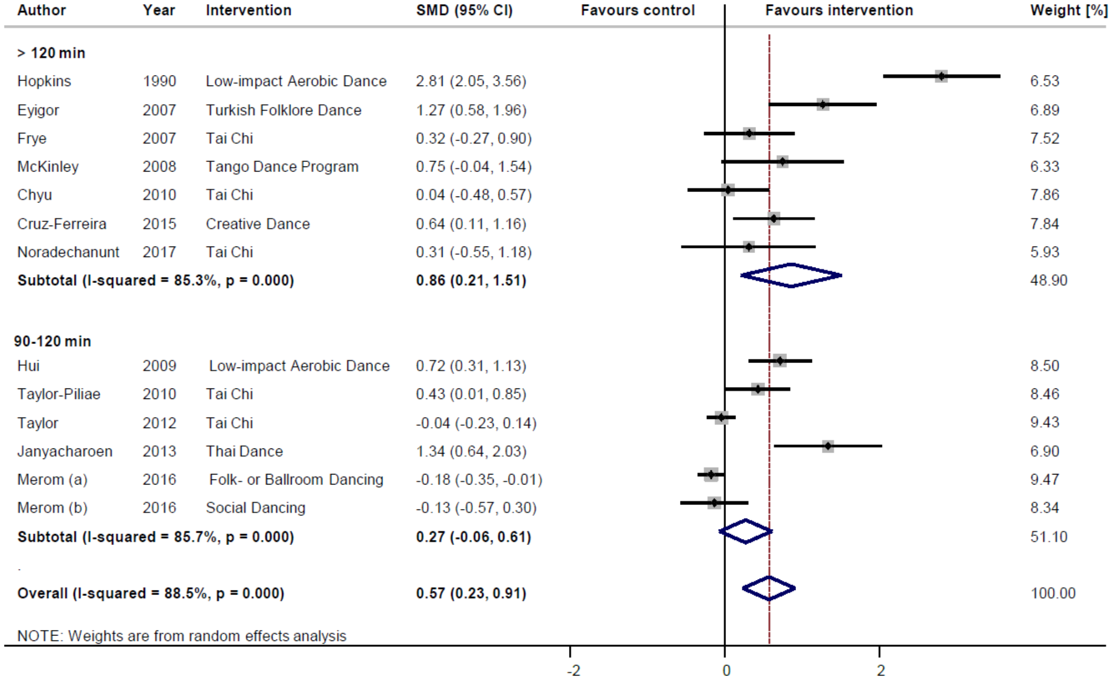

NOTE: Weights are from random effects analysis

© 2020 Mattle M et al. *JAMA Network Open.*

Sensitivity Analyses Secondary Outcomes

Association of dance-based mind-motor activities with balance
Sensitivity analysis 1: without Hopkins et al.

Author	Year	Intervention	SMD (95% CI)	Weight [%]
Choi	2004	Tai Chi with music	0.61 (0.24, 0.97)	7.61
Li F.	2005	Tai Chi	1.01 (0.91, 1.12)	8.49
Eyigor	2007	Turkish Dance	0.61 (-0.04, 1.25)	6.13
Woo	2007	Tai Chi	-0.09 (-0.59, 0.41)	6.92
Li J.	2008	Tai Chi	0.91 (0.54, 1.28)	7.58
Pereira	2008	Tai Chi	1.02 (0.55, 1.49)	7.08
Logghe	2009	Tai Chi	-0.04 (-0.27, 0.20)	8.14
Huang	2010	Tai Chi	0.83 (0.36, 1.29)	7.09
Taylor-Piliae	2010	Tai Chi	0.26 (-0.03, 0.56)	7.92
Trombetti	2011	Dalcroze Eurythmics	0.49 (0.15, 0.83)	7.72
Alves	2013	Ballroom Dance	0.49 (-0.07, 1.04)	6.63
Sun	2015	Tai Chi	-0.12 (-0.45, 0.21)	7.76
Serrano-Guzman	2016	Flamenco and Sevillanas	2.47 (1.76, 3.19)	5.75
Bennett	2018	Line Dancing	0.83 (0.01, 1.66)	5.19
**Overall (I-squared = 90.7%, p = 0.000)**			**0.63 (0.33, 0.93)**	100.00

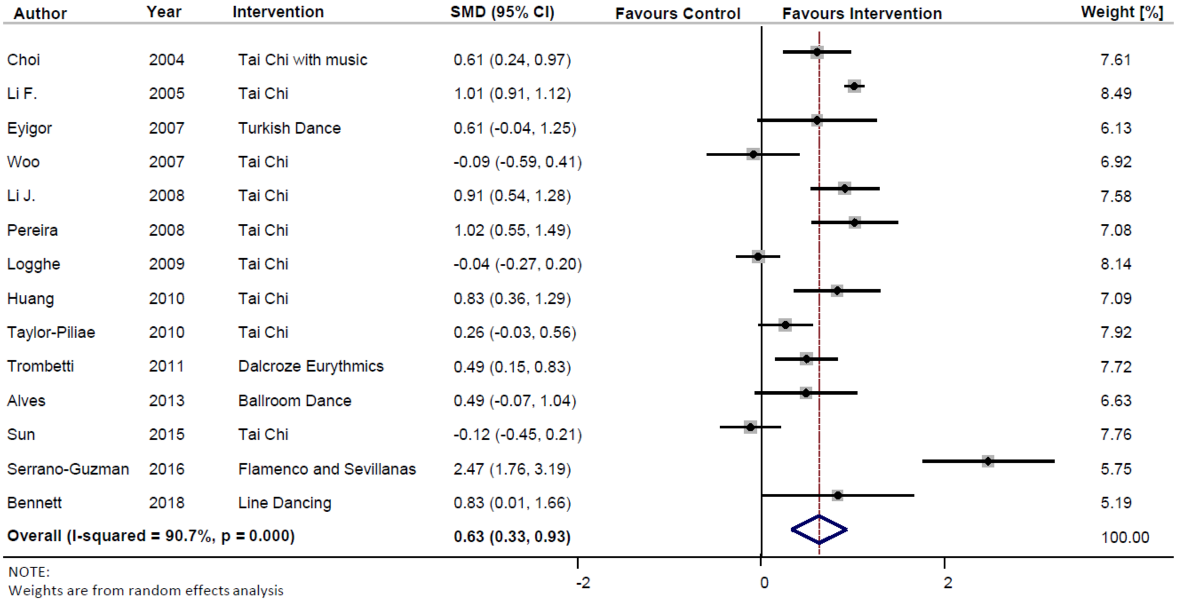

NOTE:
Weights are from random effects analysis
Total sample size by pooling 14 studies, n = 1423
Included assessments are Berg Balance Scale (BBS), One Leg Stand (OLS), and Functional Reach (FR)
Effect sizes are Hedges' *g* standardized mean differences (SMD)

Association of dance-based mind-motor activities with balance
Sensitivity analysis 2: without Serrano-Guzmann et al.

Author	Year	Intervention	SMD (95% CI)	Weight [%]
Hopkins	1990	low-impact Aerobic Dance	0.48 (-0.06, 1.02)	6.52
Choi	2004	Tai Chi with music	0.61 (0.24, 0.97)	7.60
Li F.	2005	Tai Chi	1.01 (0.91, 1.12)	8.68
Eyigor	2007	Turkish Dance	0.61 (-0.04, 1.25)	5.88
Woo	2007	Tai Chi	-0.09 (-0.59, 0.41)	6.78
Li J.	2008	Tai Chi	0.91 (0.54, 1.28)	7.56
Pereira	2008	Tai Chi	1.02 (0.55, 1.49)	6.97
Logghe	2009	Tai Chi	-0.04 (-0.27, 0.20)	8.24
Huang	2010	Tai Chi	0.83 (0.36, 1.29)	6.99
Taylor-Piliae	2010	Tai Chi	0.26 (-0.03, 0.56)	7.97
Trombetti	2011	Dalcroze Eurythmics	0.49 (0.15, 0.83)	7.72
Alves	2013	Ballroom Dance	0.49 (-0.07, 1.04)	6.45
Sun	2015	Tai Chi	-0.12 (-0.45, 0.21)	7.78
Bennett	2018	Line Dancing	0.83 (0.01, 1.66)	4.87
**Overall (I-squared = 88.9%, p = 0.000)**			**0.51 (0.24, 0.78)**	100.00

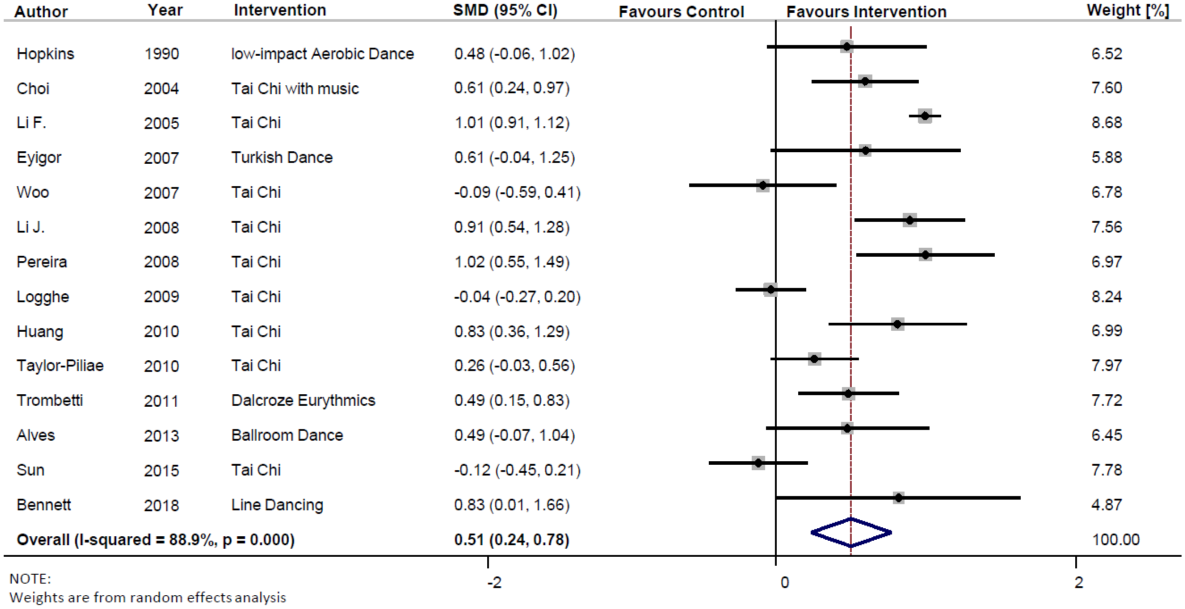

NOTE:
Weights are from random effects analysis
Total sample size by pooling 14 studies, n = 1424
Included assessments are Berg Balance Scale (BBS), One Leg Stand (OLS), and Functional Reach (FR)
Effect sizes are Hedges' *g* standardized mean differences (SMD)

© 2020 Mattle M et al. *JAMA Network Open.*

Association of dance-based mind-motor activities with mobility
Sensitivity analysis 1: without Hopkins et al.

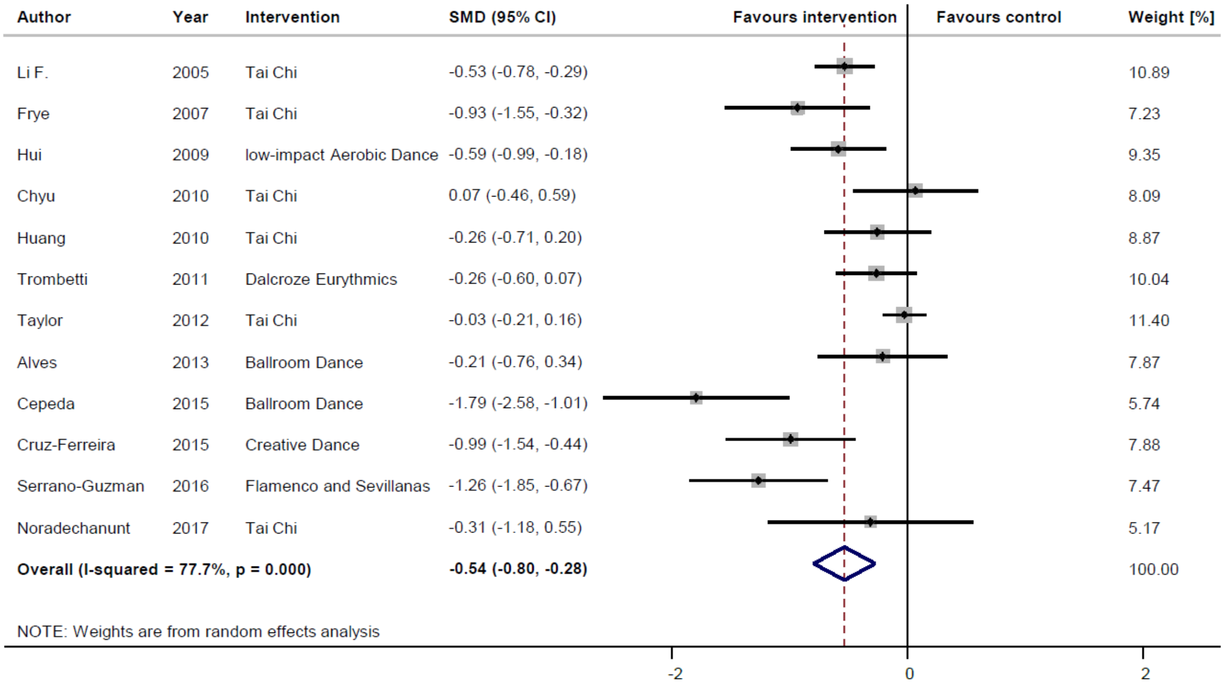

Association of dance-based mind-motor activities with mobility
Sensitivity analysis 2: without Cepeda et al.

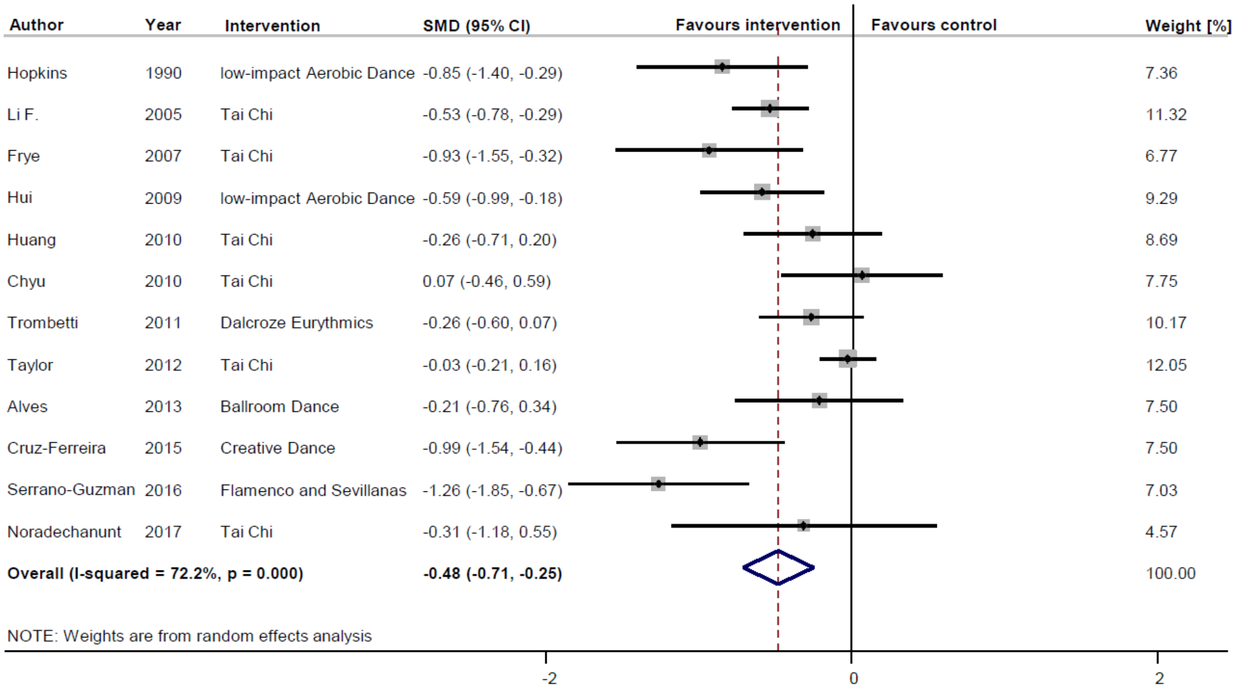

© 2020 Mattle M et al. *JAMA Network Open.*

Association of dance-based mind-motor activities with LBS
Sensitivity analysis 1: without Hopkins et al.

Author	Year	Intervention	SMD (95% CI)	Weight [%]
Eyigor	2007	Turkish Folklore Dance	1.27 (0.58, 1.96)	6.68
Frye	2007	Tai Chi	0.32 (-0.27, 0.90)	7.69
McKinley	2008	Adapted Tango	0.75 (-0.04, 1.54)	5.88
Hui	2009	Aerobic Dance	0.72 (0.31, 1.13)	9.48
Chyu	2010	Tai Chi	0.04 (-0.48, 0.57)	8.27
Taylor-Piliae	2010	Tai Chi	0.43 (0.01, 0.85)	9.40
Taylor	2012	Tai Chi	-0.04 (-0.23, 0.14)	11.53
Janyacharoen	2013	Thai Dance	1.34 (0.64, 2.03)	6.70
Cruz-Ferreira	2015	Creative Dance	0.64 (0.11, 1.16)	8.24
Merom (a)	2016	Social Dancing	-0.18 (-0.35, -0.01)	11.61
Merom (b)	2016	Ballroom Dancing	-0.13 (-0.57, 0.30)	9.18
Noradechanunt		Tai Chi	0.31 (-0.55, 1.18)	5.34
**Overall (I-squared = 80.1%, p = 0.000)**			**0.39 (0.12, 0.65)**	100.00

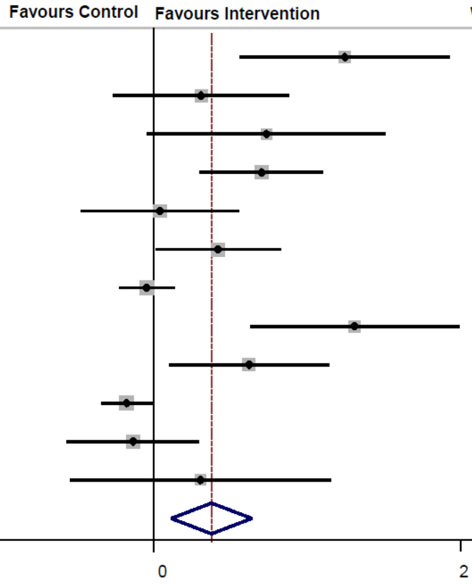

NOTE:
Weights are from random effects analysis
LBS = Lower Body Strength; Total sample size by pooling 12 studies, n = 1560
Included assessments are Sit to Stand Tests (STS): 5x STS assesses time needed to complete 5 stands, 30sec STS assesses how many stands can be competed within 30 sec
Effect sizes are Hedges' *g* standardized mean differences (SMD)
Effect sizes of 5x STS were multiplied by (-1) to achieve same direction signaling improvement as effect sizes of 30sec STS

© 2020 Mattle M et al. *JAMA Network Open.*
